# Improving core facility service discovery with an AI assistant grounded in institutional web content

**DOI:** 10.7171/001c.162898

**Published:** 2026-06-27

**Authors:** Eviatar Weizman, Dan Ben-Avraham, Robert Fluhr

**Affiliations:** 1 Ilana and Pascal Mantoux Institute for Bioinformatics Weizmann Institute of Science; 2 Plant and Environmental Sciences Weizmann Institute of Science

**Keywords:** AI, Chatbot, core facilities, RAG

## Abstract

Core facilities source advanced technologies and expertise but can remain under-utilized because researchers, students and early-career scientists, struggle to identify relevant units or cannot articulate appropriate technical inquiries. To enhance access to users, we implemented a domain grounded conversational application based on retrieval augmented generation (RAG). It combines advanced general AI-chat behavior with restricted alignment to core-facility services by uniting a Google Gemini File Search as a structured knowledge base and a Perplexity powered web agent for conceptual scientific queries. The system workflow constrains the agents to institutional domains and routes specific operational questions to a file search knowledge base. As a result, it relies on existing core facility websites and is updated in line with them. This article describes the design of the workflow, how the system is optimized including necessary guardrails to prevent general purpose chat. It proposes evaluation metrics such as veracity, cost per interaction, latency, and examples of usage. The chatbot can help researchers better define their experimental needs, discover relevant facilities they had not previously considered, and thereby increase the visibility and accessibility of institutional research infrastructures.

## Introduction

Public research institutions host numerous scientific service units or cores, each of which provide state-of-the-art technologies and expert guidance. The cores centralize specialized instruments, employ expert staff, and provide important standard operating procedures. Such shared experimental facilities are thought to optimize the use of strategic science funding.^1,2^ This has created complex operational entities that require special leadership structures and funding engines to help them thrive.^3^ Indeed, the need to optimize limited resources has led to further supra-organization of cores by connecting multiple institutions.^4,5^

The efficient use of cores is paramount and essential to justify a cores existence. However, users, whether internal or external and in academia or industry, often do not have easy access and are not always aware of the full capabilities of the cores technologies. For example, universities are populated with short-term students and postdoctoral candidates that make up a significant part of their research base. Those researchers may be unsure of which units are appropriate for a given project or unfamiliar with the terminology needed to formulate effective requests. In addition, early-career or new researchers may be unaware of the breadth of available services. As a result, opportunities for cross-unit collaboration or optimal technology choice may be missed, and core staff must repeatedly answer basic navigational questions. Similar challenges of discoverability and navigation have been noted in higher-education chatbots deployments where complex institutional structures limit the users’ ability to locate relevant services without expert mediation.^6^

In contrast to the lack on AI-assisted access to cores, user engagement improved by AI technology is well developed in the consumer field and prominent in E-commerce platforms.^7^ These systems typically use natural-language processing (NLP) as the user interface where user queries are processed by NLP components, and the system subsequently generates natural-language responses.^8^ However, such large language models (LLMs) can hallucinate, which means the models produce plausible sounding but unfaithful outputs. As a result, those systems include verification and mitigation checks, since even low error rates can be unacceptable.^9^

The key challenge is to constrain the capability of LLMs systematically to institution-specific core facility knowledge bases so that answers to queries align with its specific equipment, services, and policies. Without such focus, even very capable models tend to default to generic domain knowledge, which can misrepresent local capabilities and mislead users. Thus, to be useful, AI searches need to be restricted to a curated database that reflects the institution’s own instruments, standard operating procedures, and data-handling policies.

Generative AI systems, and retrieval-augmented generation (RAG) in particular, have been proposed as a way to mediate access to complex information ecosystems by providing natural language interfaces whose outputs are grounded in curated external sources.^10,11^ We therefore sought to design a conversational assistant that is both knowledgeable and tightly grounded in its scope for core facility services. The system, developed and tested in two large core facilities at the Weizmann Institute of Science, is intended to assist more than 1000 local life sciences researchers. The core facilities, run by over 140 technologists, combine 25 distinct units across diverse areas, including antibodies, genomics, proteomics, flow cytometry, metabolomics, imaging, bioinformatics, and drug discovery. Each unit maintains a dedicated website describing its services, instruments, workflows, and contact details. Together, these sites constitute the authoritative public documentation for the core facilities and are updated regularly by facility staff.

Our design goal was to build an AI assistant that “reads” these sites, indexes their content into a searchable knowledge base, and exposes the resulting capabilities through a web-embedded chat interface linked from the core facilities portal. The chat interface is not intended to replace interactions with facility technologists; rather, it is designed to broaden discovery of relevant technologies and to direct users to appropriate core staff. We further describe how running LLM models in parallel can provide a basis for quantitatively evaluating and monitoring system performance.

## Material and Methods

### General

The application is implemented as an n8n workflow (workflow automation n8n, version 2026, https://n8n.io/) that integrates three main components: (1) Gemini API File Search tool enabling RAG, version November 5, 2025, as a structured store of “scraped” core-facility content (https://ai.google.dev/gemini-api/docs/file-search); (2) A central reasoning engine that orchestrates responses and determines which tools to invoke, Gemini 3 Flash LLM, version December 2025, (https://blog.google/products-and-platforms/products/gemini/gemini-3-flash/); and (3) a Perplexity-backed tool for constrained web searches of the institutional domains, implemented via the Perplexity node in n8n (n8n, Perplexity node documentation, accessed April 6, 2026, https://docs.n8n.io/integrations/builtin/app-nodes/n8n-nodes-langchain.perplexity/). Thus, incoming chat messages are handled by an n8n LangChain-based RAG agent node configured with both a Gemini File Search–backed Knowledge Base tool and a Perplexity tool, together with a short-window memory buffer for conversational context. The workflow is connected to a public Chat Trigger webhook for live traffic from the website, while a LLM Trigger node supports development and testing runs. The complete n8n workflow export and custom scripts used to implement the application are provided as Supplementary Files S1–S8. A working example of the chatbot can be viewed at https://www.weizmann.ac.il/LS_CoreFacilities/.

### Knowledge-Base Construction with Gemini File Search

**Figure 1.**
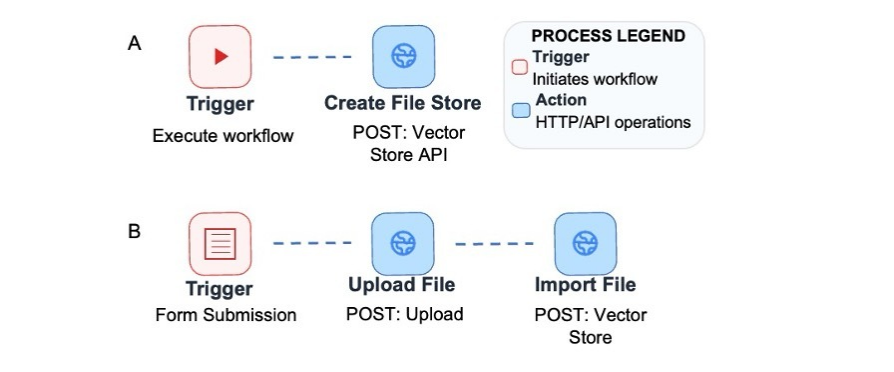
**Creation and population of the Gemini File Search Knowledge Base.** (A) Workflow for setting up the Gemini File Search document repository. In the figure, this part is shown as “Trigger - Create File Store.” The Trigger is the event that starts the process. Create File Store creates a dedicated online storage space where the documents for the Knowledge Base are kept. At the same time, this step sets up the search structure that allows the system to find information by meaning and context, rather than only by exact word matches. This is done by sending an HTTP POST request to the Google Generative Language (File Search) API: API means a software interface that allows one program to communicate with another. In return, the system provides a store identifier, which is simply a unique ID number or name for that file store. This identifier is then used by later steps in the workflow. Once the store is created, the file search service automatically manages file storage, chunking (splitting documents into smaller, manageable sections), embedding generation (turning text into numerical representations so the computer can compare passages by meaning), and vector indexing (organizing those numerical representations so relevant content can be found quickly). (B) Workflow for adding documents to the Knowledge Base. In the figure, this part is shown as “Trigger (Form Submission) - Upload File (POST: Upload) - Import File (POST: Vector Store).” Here, Trigger (Form Submission) means that the process begins when a user fills in and submits a web form. Upload File (POST: Upload) means the selected file is sent to the system using a standard web transfer method called POST. Import File (POST: Vector Store) means that the uploaded file is then added into the file store and prepared so it can be searched. Together, these steps create an automatic pipeline for continuously updating the Knowledge Base with new or revised documents. In practical terms, this allows staff to maintain facility documentation through submitting a simple form without needing specialist knowledge of artificial intelligence or programming.

A n8n HTTP Request node was created to manage a file search store via the Google Generative Language API, which specifies a display name corresponding to the life sciences chatbot and stores the resulting store identifier for subsequent use. HTML content from the official core facility websites was exported and processed into text files, which were then uploaded to the store, thus allowing Gemini File Search to index them into semantic embeddings and document chunks (Figure 1). The store contains unit descriptions, service lists, sample submission instructions, and contact information as published on the institutional websites, and all pages were accessed from publicly available institutional domains with prior approval from the institutional core facilities administration. Because the store is managed through API calls, the contents can be refreshed programmatically. In the current deployment, we perform a full reindexing approximately every three months or after major website updates as coordinated with core facility staff.

The files included in the node are described as follows. The n8n is a workflow automation platform that links different software steps together automatically to complete n8n chatbot workflow Gemini File Search (Gemini File Search chatbot.json; Supplementary File S1). Complete n8n workflows are used to create and upload files to the Google search file store (Gemini File Search template.json; Supplementary File S2). A Python script is used to scrap all webpages per unit (crawl_and_scrap_full_website_per_unit.py; Supplementary File S3). Supported file types include PDF, DOCX, TXT, JSON, and common programming-language files. Once uploaded, these files are automatically processed by Gemini File Search. This includes chunking or breaking the text into smaller parts, embedding computation or converting the text into numbers that help the system recognize related ideas, and indexing or organizing the processed content so it can be searched efficiently without manual intervention. Standalone versions of workflows are provided in the supplementary materials as Python scripts:

- create_store.py for creating a new file store (Supplementary File S4),
- upload_dir_to_store.py for uploading all files from a local folder (Supplementary File S5), and
- update_file_in_store.py for updating individual files directly from a workstation (Supplementary File S6).

A Python script is used to list all the content in the Google file store (list_store_docs.py; Supplementary File S7).

### Agent Design and Routing Logic

**Figure 2.**
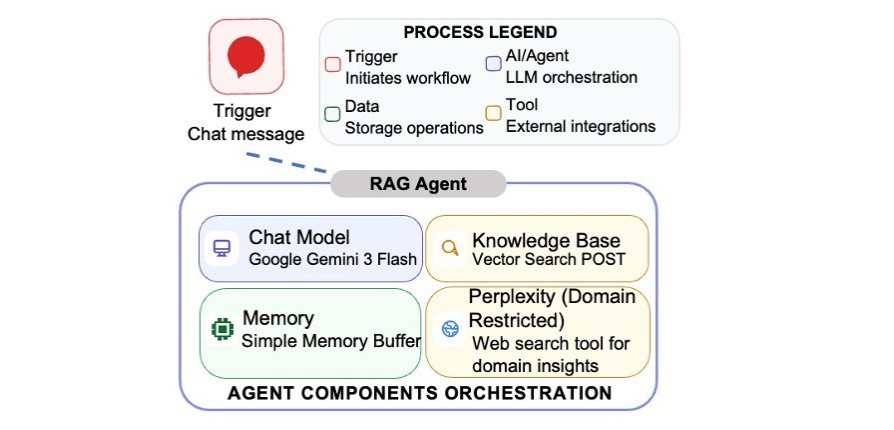
**Live query routing by a RAG-style chatbot agent.** This figure illustrates how incoming user queries are processed in the chatbot workflow. A Trigger chat message node receives the user’s message and forwards it to the RAG agent, which is a RAG agent that can combine text generation with information retrieval from connected data sources.

At the core of the system is a LangChain-style agent configured in n8n with Gemini 3 Flash as the language model and two tools: “Knowledge Base” tool backed by Gemini File Search and “Perplexity Tool” implemented as a custom node calling the Perplexity API (Figure 2). The agent is given a system message that tightly defines its role as a focused research assistant for the chosen core facilities whose only task is to help users find the appropriate unit and understand how to use it. It is granted access to the internal “Knowledge Base,” which contains official information derived from institutional websites, and to the “Perplexity Tool,” which is reserved for scientific or theoretical questions not fully addressed in the Knowledge Base. The agent is instructed to use the Knowledge Base for all questions about specific units, services, ordering procedures, sample requirements, and operational policies and to invoke the Perplexity tool only for high-level scientific explanations or conceptual comparisons. By design, while both tools might be relevant, the agent prioritizes the Knowledge Base to ensure that responses are grounded in the local context and may then optionally enrich the answer with a concise theoretical explanation from Perplexity.

Within the RAG agent, four components are shown: Chat Model (Google Gemini 3 Flash), Memory (Simple Memory Buffer), Knowledge Base, and Perplexity (Domain Restricted) web search tools for domain insights. The Chat Model provides the main response-generation capability. Memory retains a limited number of recent conversations so the system can respond in context. The Knowledge Base retrieves institution-specific information from curated internal resources. The Perplexity web search tool for domain insights is used selectively to supply broader background or scientific context but is domain restricted. Together, these components allow the chatbot to answer user queries using both conversational context and relevant retrieved information. The full n8n workflow is provided in Supplementary File S1.

### Guardrails Against General-Purpose Chat

To prevent the system from degenerating into a general-purpose chatbot, several guardrails were implemented. First, the system prompt explicitly forbids answering questions unrelated to the core facilities or the methods the facilities provide and instructs the agent to politely refuse and redirect users to facility-relevant topics when necessary. Second, the Perplexity tool is configured with domain filters that restrict search results to institutional core facility websites and a recency filter that limits retrieval to content indexed within the last 30 days, thereby reducing the risk of pulling arbitrary or outdated web material. Third, the model is instructed not to provide opinions, news, or casual conversation and to focus instead on practical next steps, such as identifying relevant units, describing services, and explaining how to initiate a project. The agent is told to state explicitly when no information is found in the tools and to avoid inventing services, equipment, or contact details. In the current configuration, the system is constrained from providing pricing or eligibility, and the user is instructed to consult and coordinate with core staff.

### Direct User Interaction (User-Facing Behavior) and Response Style

The Gemini Chat Model node is configured with a low-to-moderate “temperature,” meaning that the model’s responses are made more deterministic and consistent with limited randomness in wording while still allowing some variation in phrasing. The system message prescribes a neutral expert tone, short paragraphs and lists, and the use of headers and tables for comparisons. When mentioning units, the agent is instructed to append standardized unit names and provide URLs from the Knowledge Base as markdown links rather than internal document identifiers. If contact details or URLs are not available in the retrieved content, the agent is instructed to state this explicitly and suggest visiting the general core facility’s website or contacting central administration.

## Results

### Initial Training of the System

**Figure 3.**
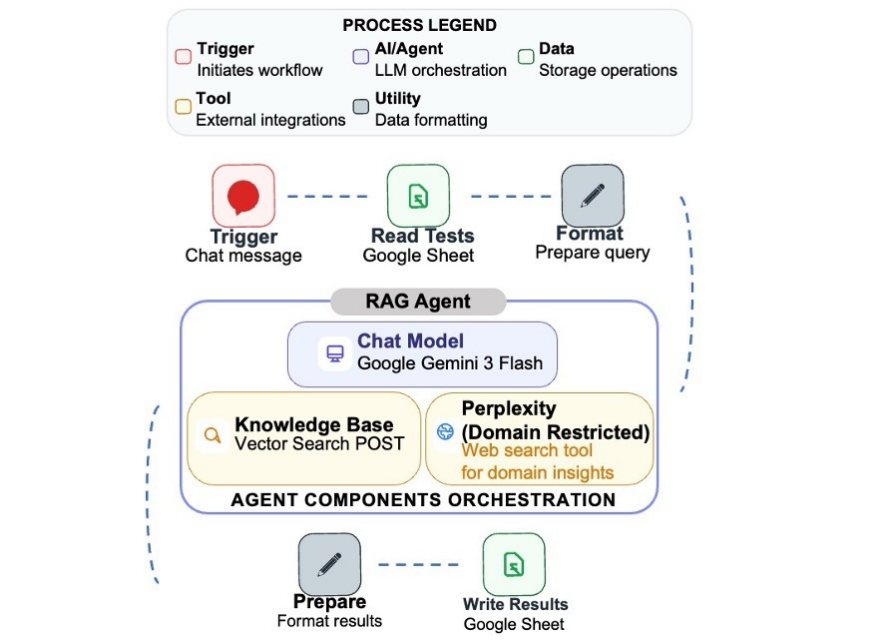
**Automated n8n workflow for testing model configurations.** A “Test Models” workflow in n8n was used to compare different Gemini–Perplexity agent configurations of the chatbot system using the same set of test questions. The workflow starts with Trigger (Chat message), reads the questions from a Google Sheet in Read Tests, and standardizes them in Format (Prepare query). The queries are then passed to a RAG agent, which combines the Chat Model (Google Gemini 3 Flash) with two external tools under Agent Components Orchestration: Knowledge Base (Vector Search POST) and Perplexity (Domain Restricted), a web search tool for domain insights. The generated outputs are processed in Prepare (Format results) and stored in a Google Sheet through Write Results, allowing systematic comparison of response quality, retrieval behavior, completeness, and latency across model configurations. The full n8n implementation is included in Supplementary File S1.

Many different and compatible agents are available, but each have functional differences. To optimize the system, several agent configurations were tested. The workflow was executed for four model configurations: (1) Gemini-2.5-Flash LLM version June 17, 2025, https://ai.google.dev/gemini-api/docs/models; (2) Sonar, Perplexity AI LLM version January 21, 2025, https://www.perplexity.ai; (3) Gemini-2.5-Flash-Lite LLM version July 22, 2025, https://ai.google.dev/gemini-api/docs/models; and (4) Gemini-3-Flash version December 17, 2025, https://blog.google/products-and-platforms/products/gemini/gemini-3-flash/ (Figure 3).

The configurations were evaluated by an automated testing workflow in n8n (see Materials and Methods) that invokes the same agent tools used in the chat but without retaining conversational memory, thus ensuring that each answer is evaluated independently of previous turns. A fixed set of 25 prompts was composed to cover routing, capability, policy, sample-preparation questions, stress-test workflows, and high-level questions intended to trigger the Perplexity tool (Supplementary File S9). For each configuration, responses to all 25 prompts were manually rated by a human expert by four criteria: correctness and factual grounding, relevance and routing to cores/contacts, completeness and usefulness, and style and clarity. Each were graded on a 0–10 scale (Supplementary File S10). Scores on each dimension were summed across the 25 prompts, yielding a possible range of 0–250 points per criteria, as reported in Table 1. Across all metrics, Gemini-3-Flash + Sonar achieved the highest scores and was therefore selected as the preferred configuration for the chat system. The key point is not the particular tools used, which are likely to evolve quickly, but the general workflow that should be implemented regardless of which tools are available in the future.

**Table 1.** Evaluation of different AI search configurations

**Model Configuration**	**Style and Clarity (0–250)**	**Completeness and Usefulness (0–250)**	**Relevance and Routing (0–250)**	**Correctness and Grounding (0–250)**
Gemini-2.5-Flash + Sonar	195	185	200	196
Gemini-2.5-Flash-Lite + Sonar	188	168	186	188
Gemini-3-Flash + Sonar	225	221	248	241

### Cost and Latency Comparison Across Models

Costs are critical to evaluate and will evolve over time. Pricing estimates for each Gemini model were obtained from the current public API documentation and pricing-calculator resources, allowing conversion of token counts to an estimated cost per interaction. For each configuration, every benchmark question was asked once, and we retrieved input-token counts, response latency, and request totals directly from the usage and billing dashboards in Google AI Studio. Using the token-usage data together with the published token prices for each Gemini model, we computed approximate costs for running the 25-prompt benchmark under each setup. These usage-based calculations showed that the total cost per configuration were a few cents with Gemini-2.5-Flash-Lite + Sonar, which was the least expensive. Given the clear quality advantage of Gemini-3-Flash + Sonar in the particular manual ratings and its modest incremental cost based on the Google AI Studio billing, we selected this configuration for production deployment. As shown in Table 2, the costs of thousands of questions that may occur in a year is estimated to be in the range of hundreds of dollars.

**Table 2.** Estimated combined API costs for Gemini + Sonar during the 25-prompt benchmark

**Model**	**Total Tokens Used (Million)**		**Price Used for Estimate (USD / 1M Tokens)**		**Total Cost (USD)**
	**Gemini**	**Sonar**	**Gemini**	**Sonar**	
Gemini-2.5-Flash + Sonar	0.33	0.02	2.50	1	0.845
Gemini-2.5-Flash-Lite + Sonar	0.27	0.015	0.40	1	0.123
Gemini-3-Flash + Sonar	0.24	0.021	3.00	1	0.741

The evaluation reported here, based on a curated set of prompts and expert ratings, provides a controlled comparison of model configurations and is not meant to capture the full variance of real-world user queries. It was designed as an internal comparison, which is useful for benchmarking select vendors.

### The Chat Experience

The following examples demonstrate representative system responses. When queried: “My antibody is weak how can it be improved?”, the chat responded: “If your antibody is ‘weak,’ it likely means you’re looking to improve its binding affinity or overall effectiveness. The Antibody Engineering Unit can help you enhance your antibody through several specialized techniques”. The chat goes on to detail different techniques that the Unit supports including humanization, isolating new antibody through single-cell sequencing, construction of antibody-protein fusions, and bispecific antibody production (Supplementary File S11a; Q1).

In a follow-up question: “How do I measure the affinity of my antibody?”, the chat described three relevant technologies: isothermal titration microcalorimetry, microscale thermophoresis, and surface plasmon resonance. It further mentions other techniques that measure thermal stability with the caveat that those are useful but less relevant (Supplementary File S11a; Q2). Another question about measuring RNA expression directed the users to multiple technologies and provides exact data as to the amounts of nucleic acids necessary (Supplementary File S11a; Q3). In this case, the data about amounts were present in the unit’s web pages. Hence, the more data present in those pages, the more detailed the answer.

When a general question was asked about what methods one can use to sort cells, a multiparagraph answer explained the possible technologies (Supplementary File S11a; Q4). In contrast, if the user specifically wanted to use a cell-sorting machine, and states this in the question, the response concisely directed the user to the sorter types, reservation rules, and preparation requirements specific to sorters only (Supplementary File S11a; Q5). When asked about measuring metabolites in plants, the chat provided a detailed answer explaining how the resources of two different core units could be combined (Supplementary File S11a; Q6). The system’s robustness was further demonstrated with the following question: “Which core facilities support extracellular vesicle research?” The chat responded with an integrated overview of technologies relevant to the area of research (Supplementary File S11a; Q7).

Importantly, the chatbot uses a dynamically indexed knowledge base, and its response quality and reproducibility depend on several factors. These include expected idiosyncrasies of RAG systems; the completeness, reliability, and structure of the indexed knowledge base and source pages; and the phrasing of user queries. To assess this variance, identical prompts were carried out after about one month and produced variations in the answers while conveying essentially the same information (compare Supplementary File S11a and Supplementary File S11b).

## Discussion

### Benefits for Accessibility and Research Planning

The results show that a domain-grounded AI assistant can navigate a complex core-facility ecosystem. By allowing users to describe their needs in natural language and returning structured suggestions that explicitly list relevant units and next steps, the system promotes broader consideration of available services and technologies. Where appropriate, it combines services in different cores to give a broader outlook on research opportunities. For students and early-career researchers in particular, the assistant helps translate vague scientific aims into concrete service requests and appropriate workflows, which improve the alignment between research projects and institutional infrastructure. The quantitative evaluation, in which Gemini-3-Flash + Sonar achieved the highest scores across correctness, routing, completeness, and clarity, suggests that high-quality language models further increase the usefulness of such guidance for research planning. As the appropriate tools are continuously changing, undoubtedly improved modules will appear; however, the architecture in which to implement these are illustrated here.

### Balancing Breadth of Knowledge with Restrictions

A central design challenge was how to offer sufficiently rich scientific explanations without allowing the assistant to drift into unfocused, general-purpose chat. The dual-tool architecture provides a pragmatic compromise: all facility-specific information is grounded in the Gemini File Search Knowledge Base, while the Perplexity-based component is invoked only for high-level conceptual questions that remain within the methodological scope of the core facilities. Strict system prompt instructions, domain-restricted search parameters, and explicit refusal behavior for out-of-scope queries collectively reduce the risk of off-topic or misleading responses. The evaluation of high-level “stress-test” prompts, which were designed to trigger the Perplexity tool, showed that Gemini-3-Flash + Sonar was able to provide richer multiunit routing while still respecting these constraints.

### Lessons Learned for Inaugurating Similar Deployments

Several lessons may be relevant for other institutions considering implementing similar systems. First, a well-structured website for core facilities is necessary and the single most critical element. This is because the quality of RAG-based responses depends closely on the clarity and completeness of the underlying web content. Second, explicit and detailed system prompts, enumerating core units, URLs, and routing rules for specialized domains, such as metabolomic and multiomic workflows, markedly improved tool selection and response relevance in our internal testing. This reduced common errors, such as omitting obvious units or assigning instruments to the wrong core. Third, the combination of an automated evaluation workflow and human rating on multiple dimensions proved valuable. This enabled an evidence-based choice of a specific production configuration and showed specific ways the system failed, which helped us gradually improve both the prompts and the content. While the system runs autonomously, a minimum of ongoing monitoring and human-in-the-loop-correction remains essential for maintenance.

### Limitations and Future Work

This implementation has several limitations. At present, the Knowledge Base relies primarily on public, institution-sponsored web content. While this material can be rich, it does not probe internal data sources maintained within the cores. In addition, it would be of interest to link published journal articles that describe how the core technologies are used. Drawing additional insight from those sources would substantially enhance support for new experimental questions, but it would also require addressing nontrivial issues of data access, privacy, and institutional oversight.

The evaluation reported here, based on a curated set of prompts and expert ratings, provides a controlled comparison of model configurations. However, it does not capture user satisfaction or long-term effects on facility utilization. Future work could include tracking how people use the system over time, running user studies to see whether they find the system useful and trustworthy, and linking it to institutional login systems to better understand the needs of different groups of users. Furthermore, over time, analytics, structured user feedback, user studies, and longitudinal evaluation of facility utilization will determine whether the assistant meaningfully improves access to core facility information and services. Importantly, the same general design can be applied to institutes that agree to share infrastructure, making core technologies easier to access across institutions and helping create a more efficient shared-capacity network.

### Financial Support/Conflict of Interest

No conflict of interest in financial research support and research does not involve human or animal subjects.

## Supplementary Material

Supplemental File

Supplemental File

Supplemental File

Supplemental File

Supplemental File

Supplemental File

Supplemental File

Supplemental File

Supplemental File

Supplemental File

Supplemental File

Supplemental File
