# Supplementary material for: Improving core facility service discovery with an AI assistant grounded in institutional web content
Source: J Biomol Tech. 2026 Jun 27;37(2):40–9. doi: 10.7171/001c.162898 (PMC13313189; doi:10.7171/001c.162898)
Supplement: Supplemental File [file jbt_2026_37_2_162898_347717.pdf]

```
Gemini File Search Store: lscf_incpm_webchat_kb
================================================

This README documents how to create a Gemini File Search store, upload Markdown
files into it, list the stored documents, and add (re-upload) individual files.

Prerequisites
-------------
1. Python 3 installed.
2. google-genai Python SDK installed:
   pip install google-genai

3. A Gemini API key stored in a text file, for example:
   GOOGLE_API.txt

   The file should contain only the API key on a single line.

4. Markdown files to ingest, stored under:
   Output_markdown


1. Create a File Search store
-----------------------------
Script: create_store.py

Usage:

python create_store.py -name lscf_incpm_webchat_kb -api GOOGLE_API.txt

Notes:
- -name sets the display name of the File Search store.
- -api points to the text file containing the Gemini API key.
- The script prints the created store's full name, for example:
  fileSearchStores/lscfincpmwebchatkb-******

You will use this full store name in the other scripts.


2. Upload a directory of Markdown files to the store
----------------------------------------------------
Script: upload_dir_to_store.py

Usage:

python upload_dir_to_store.py -store fileSearchStores/lscfincpmwebchatkb-******
-path "H:\PATH_TO\Output_markdown" -api "H:\PATH_to\GOOGLE_API.txt"

Notes:
- -store is the full store name printed by create_store.py.
- -path is the directory containing the .md files to ingest.
```

```
- -api is the path to the API key file.
- The script iterates over all .md files in the directory, uploads each
  file into the File Search store, and waits for indexing to complete.


3. List documents currently in the File Search store
----------------------------------------------------
Script: list_store_docs.py

Usage:

python list_store_docs.py -store fileSearchStores/lscfincpmwebchatkb-****** -api
"H:\PATH_to\GOOGLE_API.txt" > lscfincpmwebchatkb-******_list_store_files.txt

Notes:
- -store is the full store name.
- -api is the path to the API key file.
- The script prints all documents in the store (including their internal
  document names and display_name values).
- The example redirects the output to:
  lscfincpmwebchatkb-cbxac8u5eq8r_list_store_files.txt


4. Add (re-upload) a single file to the store
---------------------------------------------
Script: update_file_in_store.py

Usage:

python update_file_in_store.py -store fileSearchStores/lscfincpmwebchatkb-******
-api "H:\PATH_to\GOOGLE_API.txt" -file "H:\PATH_to\filename.md"

Notes:
- -store is the full store name.
- -api is the path to the API key file.
- -file is the path to the local Markdown file to upload.
- The script currently uploads the file as a new document (it does not
  delete older versions due to limitations in the SDK's delete methods).
- The display name defaults to the filename; you can override it with -name
  if needed.


5. Using the store from a chatbot (high level)
----------------------------------------------
From your chatbot, you call the Gemini API with:
- model set to a supported model (e.g. gemini-3.0-flash)
- tools configured to include the File Search tool pointing at:
  fileSearchStores/lscfincpmwebchatkb-******

The model will then ground its answers in the documents you uploaded
```

into this store.
