# Supplementary material for: Improving core facility service discovery with an AI assistant grounded in institutional web content
Source: J Biomol Tech. 2026 Jun 27;37(2):40–9. doi: 10.7171/001c.162898 (PMC13313189; doi:10.7171/001c.162898)
Supplement: Supplemental File [file jbt_2026_37_2_162898_347718.pdf]

```python
# update_file_in_store.py
import argparse
import os
from google import genai


def parse_args():
    parser = argparse.ArgumentParser(
        description="Upload (or re-upload) a file into a Gemini File Search store."
    )
    parser.add_argument(
        "-store",
        "--store",
        required=True,
        help="Full File Search store name (e.g. fileSearchStores/xxxx).",
    )
    parser.add_argument(
        "-api",
        "--api",
        required=True,
        help="Path to a text file containing the Gemini API key.",
    )
    parser.add_argument(
        "-file",
        "--file",
        required=True,
        help="Path to the local file to upload.",
    )
    parser.add_argument(
        "-name",
        "--name",
        required=False,
        help="Display name to set for this upload. "
             "Defaults to the file's basename.",
    )
    return parser.parse_args()


def read_api_key(path: str) -> str:
    with open(path, "r", encoding="utf-8") as f:
        return f.read().strip()


def main():
    args = parse_args()

    store_name = args.store
    api_key_path = args.api
    file_path = args.file
```

```python
    if not os.path.isfile(file_path):
        raise SystemExit(f"File does not exist: {file_path}")

    display_name = args.name or os.path.basename(file_path)

    api_key = read_api_key(api_key_path)
    client = genai.Client(api_key=api_key)

    print(f"Uploading file as new document: {file_path}")
    op = client.file_search_stores.upload_to_file_search_store(
        file_search_store_name=store_name,
        file=file_path,
        config={
            "display_name": display_name,
            "chunking_config": {
                "white_space_config": {
                    "max_tokens_per_chunk": 200,
                    "max_overlap_tokens": 20,
                }
            },
        },
    )
    op = client.operations.get(op)
    print("Upload completed.")


if __name__ == "__main__":
    main()
```
