# Supplementary material for: Improving core facility service discovery with an AI assistant grounded in institutional web content
Source: J Biomol Tech. 2026 Jun 27;37(2):40–9. doi: 10.7171/001c.162898 (PMC13313189; doi:10.7171/001c.162898)
Supplement: Supplemental File [file jbt_2026_37_2_162898_347719.pdf]

```
from pathlib import Path
from firecrawl import Firecrawl
import json
import re

def safe_filename(name, max_len=50):
    """Safe Windows filenames"""
    name = re.sub(r'[<>:"/\\|?*]', '_', name)
    name = re.sub(r'\s+', '_', name.strip())
    return name[:max_len]

firecrawl = Firecrawl(api_key=Path("firecrawl_api.txt").read_text().strip())


# YOUR DICT: unit → [exact urls from sitemap]
UNIT_URLS = {
    "G-INCPM_Home": [
        "https://g-incpm.weizmann.ac.il/",
        "https://g-incpm.weizmann.ac.il/node/1",
        "https://g-incpm.weizmann.ac.il/about/steering-committee",
        "https://g-incpm.weizmann.ac.il/about/citing-us",
        "https://g-incpm.weizmann.ac.il/supporters",
        "https://g-incpm.weizmann.ac.il/operations-center/administration",
        "https://g-incpm.weizmann.ac.il/outside-users-rebates"
    ],
    "Crown Institute for Genomics": [
        "https://g-incpm.weizmann.ac.il/units/CrownGenomics/about",
        "https://g-incpm.weizmann.ac.il/units/CrownGenomics/supporters",
        "https://g-incpm.weizmann.ac.il/units/CrownGenomics/our-services",
        "https://g-incpm.weizmann.ac.il/units/CrownGenomics/equipment",
        "https://g-incpm.weizmann.ac.il/units/CrownGenomics/safety-guidelines",
        "https://g-incpm.weizmann.ac.il/units/CrownGenomics/scientific_activities",

"https://g-incpm.weizmann.ac.il/units/CrownGenomics/scientific-publications",

"https://g-incpm.weizmann.ac.il/units/crown-institute-genomics/crown-genomics-team"
,
        "https://g-incpm.weizmann.ac.il/units/CrownGenomics/what%27s-new",
        "https://g-incpm.weizmann.ac.il/units/CrownGenomics/contact",
        "https://g-incpm.weizmann.ac.il/units/CrownGenomics/sample-submission",
        "https://g-incpm.weizmann.ac.il/node/197",
        "https://g-incpm.weizmann.ac.il/node/198",
        "https://g-incpm.weizmann.ac.il/node/199",
        "https://g-incpm.weizmann.ac.il/node/200"
    ],
    "Ilana And Pascal Mantoux Institute for Bioinformatics": [
        "https://g-incpm.weizmann.ac.il/units/MantouxBioinformatics/about",
        "https://g-incpm.weizmann.ac.il/units/MantouxBioinformatics/supporters",

"https://g-incpm.weizmann.ac.il/units/MantouxBioinformatics/scientific_activities",
```

"https://g-incpm.weizmann.ac.il/units/MantouxBioinformatics/scientific-publications
",
        "https://g-incpm.weizmann.ac.il/units/MantouxBioinformatics/team",
        "https://g-incpm.weizmann.ac.il/units/MantouxBioinformatics/what%27s-new",
        "https://g-incpm.weizmann.ac.il/units/MantouxBioinformatics/contact",

"https://g-incpm.weizmann.ac.il/units/MantouxBioinformatics/bioinformatics-courses"
    ],
    "de Botton Institute for Protein Profiling": [
        "https://g-incpm.weizmann.ac.il/units/deBottonProteinProfiling/about",
        "https://g-incpm.weizmann.ac.il/units/deBottonProteinProfiling/supporters",
        "https://g-incpm.weizmann.ac.il/units/deBottonProteinProfiling/equipment",

"https://g-incpm.weizmann.ac.il/units/deBottonProteinProfiling/safety-guidelines",
        "https://g-incpm.weizmann.ac.il/units/deBottonProteinProfiling/dips",
        "https://g-incpm.weizmann.ac.il/units/deBottonProteinProfiling/rawbeans",

"https://g-incpm.weizmann.ac.il/units/deBottonProteinProfiling/publications",
        "https://g-incpm.weizmann.ac.il/units/deBottonProteinProfiling/highlights",

"https://g-incpm.weizmann.ac.il/units/deBottonProteinProfiling/useful-links",
        "https://g-incpm.weizmann.ac.il/units/deBottonProteinProfiling/team",

"https://g-incpm.weizmann.ac.il/units/deBottonProteinProfiling/what%27s-new",

"https://g-incpm.weizmann.ac.il/units/deBottonProteinProfiling/protein-profiling-co
urses",
        "https://g-incpm.weizmann.ac.il/units/deBottonProteinProfiling/contact",

"https://g-incpm.weizmann.ac.il/units/deBottonProteinProfiling/sample-submission",

"https://g-incpm.weizmann.ac.il/units/deBottonProteinProfiling/co-ip-and-pulldown",
        "https://g-incpm.weizmann.ac.il/node/120",
        "https://g-incpm.weizmann.ac.il/whole-celltissue-lysate",
        "https://g-incpm.weizmann.ac.il/biofluids"

    ],
    "David and Fela Shapell Family Institute for Preclinical Studies": [
        "https://g-incpm.weizmann.ac.il/units/ShapellPreclinical/about",
        "https://g-incpm.weizmann.ac.il/units/ShapellPreclinical/supporters",

"https://g-incpm.weizmann.ac.il/units/ShapellPreclinical/scientific_activities",
        "https://g-incpm.weizmann.ac.il/units/ShapellPreclinical/contact"

    ],
    "Maurice And Vivienne Wohl Institute for Drug Discovery": [
        "https://g-incpm.weizmann.ac.il/units/WohlDrugDiscovery/about",
        "https://g-incpm.weizmann.ac.il/units/WohlDrugDiscovery/supporters",
        "https://g-incpm.weizmann.ac.il/units/WohlDrugDiscovery/our-services",

```
        "https://g-incpm.weizmann.ac.il/units/WohlDrugDiscovery/assays",
        "https://g-incpm.weizmann.ac.il/units/WohlDrugDiscovery/equipment",
        "https://g-incpm.weizmann.ac.il/units/WohlDrugDiscovery/rna-therapeutics-",
        "https://g-incpm.weizmann.ac.il/units/WohlDrugDiscovery/safety-guidelines",

"https://g-incpm.weizmann.ac.il/units/WohlDrugDiscovery/chemical-libraries",

"https://g-incpm.weizmann.ac.il/units/WohlDrugDiscovery/scientific_activities",
        "https://g-incpm.weizmann.ac.il/units/WohlDrugDiscovery/publications",
        "https://g-incpm.weizmann.ac.il/units/WohlDrugDiscovery/team",
        "https://g-incpm.weizmann.ac.il/units/WohlDrugDiscovery/what%27s-new",
        "https://g-incpm.weizmann.ac.il/units/WohlDrugDiscovery/contact",

"https://g-incpm.weizmann.ac.il/units/WohlDrugDiscovery/drug-discovery-courses"
    ],
    "Medicinal Chemistry": [
        "https://g-incpm.weizmann.ac.il/units/MedicinalChemistry/about",
        "https://g-incpm.weizmann.ac.il/units/MedicinalChemistry/our-services",
        "https://g-incpm.weizmann.ac.il/units/MedicinalChemistry/equipment",

"https://g-incpm.weizmann.ac.il/units/MedicinalChemistry/safety-guidelines",

"https://g-incpm.weizmann.ac.il/units/medicinal-chemistry/peptide-synthesis",

"https://g-incpm.weizmann.ac.il/units/MedicinalChemistry/chemical-libraries",
        "https://g-incpm.weizmann.ac.il/units/MedicinalChemistry/team",
        "https://g-incpm.weizmann.ac.il/units/MedicinalChemistry/what%27s-new",
        "https://g-incpm.weizmann.ac.il/units/MedicinalChemistry/contact"
    ],
    "Information Technology": [
        "https://g-incpm.weizmann.ac.il/units/InformationTechnology/about",
        "https://g-incpm.weizmann.ac.il/units/InformationTechnology/our-services",
        "https://g-incpm.weizmann.ac.il/units/InformationTechnology/equipment",

"https://g-incpm.weizmann.ac.il/units/InformationTechnology/scientific_activities",
        "https://g-incpm.weizmann.ac.il/units/InformationTechnology/team",
        "https://g-incpm.weizmann.ac.il/units/InformationTechnology/contact"

    ],
    "LSCF Home": [
        "https://www.weizmann.ac.il/LS_CoreFacilities/",
        "https://www.weizmann.ac.il/LS_CoreFacilities/directory",
        "https://www.weizmann.ac.il/LS_CoreFacilities/outside-users-rebates",
        "https://www.weizmann.ac.il/LS_CoreFacilities/administration",
        "https://www.weizmann.ac.il/LS_CoreFacilities/new-technologies"
    ],
    "Advanced Sequencing Technlogies": [

"https://www.weizmann.ac.il/LS_CoreFacilities/advanced-sequencing-technlogies/about
",
```

"https://www.weizmann.ac.il/LS_CoreFacilities/advanced-sequencing-technlogies/how-order",

"https://www.weizmann.ac.il/LS_CoreFacilities/advanced-sequencing-technlogies/publications",

"https://www.weizmann.ac.il/LS_CoreFacilities/advanced-sequencing-technlogies/support-and-materials",

"https://www.weizmann.ac.il/LS_CoreFacilities/advanced-sequencing-technlogies/staff-and-contacts"
    ],
    "Antibody Engineering": [
        "https://www.weizmann.ac.il/LS_CoreFacilities/antibody/about",

"https://www.weizmann.ac.il/LS_CoreFacilities/antibody-engineering/how-order",
        "https://www.weizmann.ac.il/LS_CoreFacilities/antibody/publications",

"https://www.weizmann.ac.il/LS_CoreFacilities/antibody-engineering/staff-and-contacts",

"https://www.weizmann.ac.il/LS_CoreFacilities/antibody-engineering/education"
    ],
    "Bacteriology Genomic Repository": [

"https://www.weizmann.ac.il/LS_CoreFacilities/bacteriology-genomic-repository/about",

"https://www.weizmann.ac.il/LS_CoreFacilities/bacteriology-genomic-repository/how-order",

"https://www.weizmann.ac.il/LS_CoreFacilities/bacteriology-genomic-repository/publications",

"https://www.weizmann.ac.il/LS_CoreFacilities/bacteriology-genomic-repository/resources",

"https://www.weizmann.ac.il/LS_CoreFacilities/bacteriology-genomic-repository/safety-0",

"https://www.weizmann.ac.il/LS_CoreFacilities/bacteriology-genomic-repository/staff-and-contacts",

"https://www.weizmann.ac.il/LS_CoreFacilities/bacteriology-genomic-repository/support-0",

"https://www.weizmann.ac.il/LS_CoreFacilities/bacteriology-genomic-repository/education"
    ],

"Bioinformatics LSCF": [
"https://www.weizmann.ac.il/LS_CoreFacilities/bioinformatics-lscf/about",
"https://www.weizmann.ac.il/LS_CoreFacilities/bioinformatics-lscf/courses",

"https://www.weizmann.ac.il/LS_CoreFacilities/bioinformatics-lscf/how-order",
"https://www.weizmann.ac.il/LS_CoreFacilities/bioinformatics/publications",

"https://www.weizmann.ac.il/LS_CoreFacilities/bioinformatics-lscf/staff-and-contact
",
"https://www.weizmann.ac.il/LS_CoreFacilities/bioinformatics-lscf/support",

"https://www.weizmann.ac.il/LS_CoreFacilities/bioinformatics-lscf/education"
],
"Biomedical Research": [

"https://centers.weizmann.ac.il/GrandPersonalMedicine/units/MantouxBioinformatics/a
bout",
"https://www.weizmann.ac.il/LS_CoreFacilities/biomedical-research/about",

"https://www.weizmann.ac.il/LS_CoreFacilities/biomedical-research/how-order",

"https://www.weizmann.ac.il/LS_CoreFacilities/biomedical-research/staff-and-contact
s",

"https://www.weizmann.ac.il/LS_CoreFacilities/biomedical-research/education"
],
"Crystallisation and Structure Determination": [

"https://www.weizmann.ac.il/LS_CoreFacilities/crystallisation-and-structure-determi
nation/about",

"https://www.weizmann.ac.il/LS_CoreFacilities/crystallisation-and-structure-determi
nation/safety-0",

"https://www.weizmann.ac.il/LS_CoreFacilities/crystallization-structural-determinat
ion-and-structure-modeling/how-order",

"https://www.weizmann.ac.il/LS_CoreFacilities/crystallisation-and-structure-determi
nation/sample-preparation",

"https://www.weizmann.ac.il/LS_CoreFacilities/crystallisation-and-structure-determi
nation/resources",

"https://www.weizmann.ac.il/LS_CoreFacilities/crystallisation-and-structure-determi
nation/courses",

"https://www.weizmann.ac.il/LS_CoreFacilities/crystallisation-and-structure-determi
nation/publications",

"https://www.weizmann.ac.il/LS_CoreFacilities/crystallisation-and-structure-determi

```
nation/staff-and-contacts",

"https://www.weizmann.ac.il/LS_CoreFacilities/crystallisation-and-structure-determi
nation/education"
    ],
    "DNA Manipulation": [
        "https://www.weizmann.ac.il/LS_CoreFacilities/dna-manipulation/about",

"https://www.weizmann.ac.il/LS_CoreFacilities/dna-manipulation/safety-guidelines",
        "https://www.weizmann.ac.il/LS_CoreFacilities/dna-manipulation/how-order",
        "https://www.weizmann.ac.il/LS_CoreFacilities/dna-manipulation/courses",

"https://www.weizmann.ac.il/LS_CoreFacilities/dna-manipulation/publications",

"https://www.weizmann.ac.il/LS_CoreFacilities/dna-manipulation/staff-and-contacts",
        "https://www.weizmann.ac.il/LS_CoreFacilities/dna-manipulation/education"
    ],
    "DNA sequencing": [
        "https://www.weizmann.ac.il/LS_CoreFacilities/dna-sequencing/about",
        "https://www.weizmann.ac.il/LS_CoreFacilities/dna-sequencing/how-order",
        "https://www.weizmann.ac.il/LS_CoreFacilities/dna-sequencing/publications",

"https://www.weizmann.ac.il/LS_CoreFacilities/dna-sequencing/results-analysis",
        "https://www.weizmann.ac.il/LS_CoreFacilities/dna-sequencing/safety",

"https://www.weizmann.ac.il/LS_CoreFacilities/dna-sequencing/sample-preparation",

"https://www.weizmann.ac.il/LS_CoreFacilities/dna-sequencing/staff-and-contacts",
        "https://www.weizmann.ac.il/LS_CoreFacilities/dna-sequencing/support",
        "https://www.weizmann.ac.il/LS_CoreFacilities/dna-sequencing/education"
    ],
    "Ecosystem Microbiome Research": [

"https://www.weizmann.ac.il/LS_CoreFacilities/ecosystem-microbiome-research/about",

"https://www.weizmann.ac.il/LS_CoreFacilities/ecosystem-microbiome-research/service
s",

"https://www.weizmann.ac.il/LS_CoreFacilities/ecosystem-microbiome-research/how-ord
er",

"https://www.weizmann.ac.il/LS_CoreFacilities/ecosystem-microbiome-research/publica
tions",

"https://www.weizmann.ac.il/LS_CoreFacilities/ecosystem-microbiome-research/staff-a
nd-contacts"
    ],
    "Flow Cytometry": [
        "https://www.weizmann.ac.il/LS_CoreFacilities/flow-cytometry/about",
        "https://www.weizmann.ac.il/LS_CoreFacilities/flow-cytometry/courses",
```

"https://www.weizmann.ac.il/LS_CoreFacilities/flow-cytometry/how-order",

"https://www.weizmann.ac.il/LS_CoreFacilities/flow-cytometry/publications-0",
"https://www.weizmann.ac.il/LS_CoreFacilities/flow-cytometry/resources",
"https://www.weizmann.ac.il/LS_CoreFacilities/flow-cytometry/results",
"https://www.weizmann.ac.il/LS_CoreFacilities/flow-cytometry/safety",

"https://www.weizmann.ac.il/LS_CoreFacilities/flow-cytometry/sample-preparation",

"https://www.weizmann.ac.il/LS_CoreFacilities/flow-cytometry/staff-and-contact",
"https://www.weizmann.ac.il/LS_CoreFacilities/flow-cytometry/support",
"https://www.weizmann.ac.il/LS_CoreFacilities/flow-cytometry/education"
],
"Fly food": [
"https://www.weizmann.ac.il/LS_CoreFacilities/fly-food/about",
"https://www.weizmann.ac.il/LS_CoreFacilities/fly-food/how-order",
"https://www.weizmann.ac.il/LS_CoreFacilities/units/fly-food/resources",
"https://www.weizmann.ac.il/LS_CoreFacilities/fly-food/safety",
"https://www.weizmann.ac.il/LS_CoreFacilities/fly-food/staff-and-contact",
"https://www.weizmann.ac.il/LS_CoreFacilities/fly-food/education"
],
"Genomics, Sandbox": [
"https://www.weizmann.ac.il/LS_CoreFacilities/genomics-sandbox/about",
"https://www.weizmann.ac.il/LS_CoreFacilities/genomics-sandbox/how-order",
"https://www.weizmann.ac.il/LS_CoreFacilities/genomics-sandbox/results",

"https://www.weizmann.ac.il/LS_CoreFacilities/genomics-sandbox/sample-preparation",

"https://www.weizmann.ac.il/LS_CoreFacilities/genomics-sandbox/staff-and-contacts",

"https://www.weizmann.ac.il/LS_CoreFacilities/genomics-sandbox/safety-guidelines",
"https://www.weizmann.ac.il/LS_CoreFacilities/genomics-sandbox/education"
],
"Irradiation": [
"https://www.weizmann.ac.il/LS_CoreFacilities/irradiation/about",
"https://www.weizmann.ac.il/LS_CoreFacilities/irradiation/how-order",
"https://www.weizmann.ac.il/LS_CoreFacilities/irradiation/resources",
"https://www.weizmann.ac.il/LS_CoreFacilities/irradiation/safety",

"https://www.weizmann.ac.il/LS_CoreFacilities/irradiation/staff-and-contacts",
"https://www.weizmann.ac.il/LS_CoreFacilities/irradiation/education"
],
"Mass Cytometry": [
"https://www.weizmann.ac.il/LS_CoreFacilities/mass-cytometry/about",
"https://www.weizmann.ac.il/LS_CoreFacilities/mass-cytometry/resources",
"https://www.weizmann.ac.il/LS_CoreFacilities/mass-cytometry/how-order",
"https://www.weizmann.ac.il/LS_CoreFacilities/mass-cytometry/courses",
"https://www.weizmann.ac.il/LS_CoreFacilities/mass-cytometry/publications",
"https://www.weizmann.ac.il/LS_CoreFacilities/mass-cytometry/safety",

```
"https://www.weizmann.ac.il/LS_CoreFacilities/mass-cytometry/staff-and-contacts",
        "https://www.weizmann.ac.il/LS_CoreFacilities/mass-cytometry/education",
        "https://www.weizmann.ac.il/LS_CoreFacilities/mass-cytometry/protocols"
    ],
    "Mass spectrometry imaging": [

"https://www.weizmann.ac.il/LS_CoreFacilities/mass-spectrometry-imaging/about",

"https://www.weizmann.ac.il/LS_CoreFacilities/mass-spectrometry-imaging/how-order",

"https://www.weizmann.ac.il/LS_CoreFacilities/mass-spectrometry-imaging/resources",

"https://www.weizmann.ac.il/LS_CoreFacilities/mass-spectrometry-imaging/services",

"https://www.weizmann.ac.il/LS_CoreFacilities/mass-spectrometry-imaging/safety",

"https://www.weizmann.ac.il/LS_CoreFacilities/mass-spectrometry-imaging/publication
s",

"https://www.weizmann.ac.il/LS_CoreFacilities/mass-spectrometry-imaging/staff-and-c
ontacts",

"https://www.weizmann.ac.il/LS_CoreFacilities/mass-spectrometry-imaging/education"
    ],
    "Metabolic Profiling": [
        "https://www.weizmann.ac.il/LS_CoreFacilities/metabolic-profiling/about",

"https://www.weizmann.ac.il/LS_CoreFacilities/metabolic-profiling/how-order",

"https://www.weizmann.ac.il/LS_CoreFacilities/metabolic-profiling/publications-0",

"https://www.weizmann.ac.il/LS_CoreFacilities/units/metabolic-profiling/resources",

"https://www.weizmann.ac.il/LS_CoreFacilities/metabolic-profiling/staff-and-contact
",
        "https://www.weizmann.ac.il/LS_CoreFacilities/metabolic-profiling/safety",

"https://www.weizmann.ac.il/LS_CoreFacilities/metabolic-profiling/education"
    ],
    "MICC Cell Observatory": [
        "https://www.weizmann.ac.il/LS_CoreFacilities/micc-cell-observatory/about",

"https://www.weizmann.ac.il/LS_CoreFacilities/micc-cell-observatory/resources",

"https://www.weizmann.ac.il/LS_CoreFacilities/micc-cell-observatory/sample-preparat
ion",

"https://www.weizmann.ac.il/LS_CoreFacilities/micc-cell-observatory/support",

"https://www.weizmann.ac.il/LS_CoreFacilities/micc-cell-observatory/staff-and-conta
```

ct",

"https://www.weizmann.ac.il/LS_CoreFacilities/micc-cell-observatory/courses",

"https://www.weizmann.ac.il/LS_CoreFacilities/micc-cell-observatory/made-weizmann",

"https://www.weizmann.ac.il/LS_CoreFacilities/micc-cell-observatory/publications",

"https://www.weizmann.ac.il/LS_CoreFacilities/micc-cell-observatory/safety",

"https://www.weizmann.ac.il/LS_CoreFacilities/micc-cell-observatory/how-order",

"https://www.weizmann.ac.il/LS_CoreFacilities/micc-cell-observatory/education"
    ],
    "MRI": [
        "https://www.weizmann.ac.il/LS_CoreFacilities/mri/about",
        "https://www.weizmann.ac.il/LS_CoreFacilities/mri/how-order",
        "https://www.weizmann.ac.il/LS_CoreFacilities/mri/publications",
        "https://www.weizmann.ac.il/LS_CoreFacilities/mri/resources",
        "https://www.weizmann.ac.il/LS_CoreFacilities/mri/results",
        "https://www.weizmann.ac.il/LS_CoreFacilities/mri/safety",
        "https://www.weizmann.ac.il/LS_CoreFacilities/mri/staff-and-contact",
        "https://www.weizmann.ac.il/LS_CoreFacilities/mri/support",
        "https://www.weizmann.ac.il/LS_CoreFacilities/mri/education"
    ],
    "Protein Analysis": [
        "https://www.weizmann.ac.il/LS_CoreFacilities/protein-analysis/about"
        "https://www.weizmann.ac.il/LS_CoreFacilities/protein-analysis/how-order",

"https://www.weizmann.ac.il/LS_CoreFacilities/protein-analysis/publications",

"https://www.weizmann.ac.il/LS_CoreFacilities/units/protein-analysis/resources",
        "https://www.weizmann.ac.il/LS_CoreFacilities/protein-analysis/safety",

"https://www.weizmann.ac.il/LS_CoreFacilities/protein-analysis/sample-preparation",

"https://www.weizmann.ac.il/LS_CoreFacilities/protein-analysis/staff-and-contact",
        "https://www.weizmann.ac.il/LS_CoreFacilities/protein-analysis/education"
    ],
    "Protein Expression": [
        "https://www.weizmann.ac.il/LS_CoreFacilities/protein-expression/about",
        "https://www.weizmann.ac.il/LS_CoreFacilities/protein-expression/courses",

"https://www.weizmann.ac.il/LS_CoreFacilities/protein-expression/publications",

"https://www.weizmann.ac.il/LS_CoreFacilities/protein-expression/staff-and-contacts
",

"https://www.weizmann.ac.il/LS_CoreFacilities/protein-expression/how-order",

"https://www.weizmann.ac.il/LS_CoreFacilities/protein-expression/resources",
        "https://www.weizmann.ac.il/LS_CoreFacilities/protein-expression/safety",
        "https://www.weizmann.ac.il/LS_CoreFacilities/protein-expression/education"
    ],
    "Protein Purification": [
        "https://www.weizmann.ac.il/LS_CoreFacilities/protein-purification/about",

"https://www.weizmann.ac.il/LS_CoreFacilities/protein-purification/courses",

"https://www.weizmann.ac.il/LS_CoreFacilities/protein-purification/how-order",

"https://www.weizmann.ac.il/LS_CoreFacilities/protein-purification/publications",

"https://www.weizmann.ac.il/LS_CoreFacilities/protein-purification/resources",

"https://www.weizmann.ac.il/LS_CoreFacilities/protein-purification/staff-and-contac
ts",
        "https://www.weizmann.ac.il/LS_CoreFacilities/protein-purification/safety",

"https://www.weizmann.ac.il/LS_CoreFacilities/protein-purification/education"
    ],
    "Stem Cells & Organoids": [
        "https://www.weizmann.ac.il/LS_CoreFacilities/stem-cells-organoids/about",
        "https://www.weizmann.ac.il/LS_CoreFacilities/stem-cells-organoids/safety",

"https://www.weizmann.ac.il/LS_CoreFacilities/stem-cells-organoids/staff-and-contac
ts",

"https://www.weizmann.ac.il/LS_CoreFacilities/stem-cells-organoids/education"
    ],
    "Targeted Metabolomics": [
        "https://www.weizmann.ac.il/LS_CoreFacilities/targeted-metabolomics/about",

"https://www.weizmann.ac.il/LS_CoreFacilities/targeted-metabolomics/how-order",

"https://www.weizmann.ac.il/LS_CoreFacilities/targeted-metabolomics/publications",

"https://www.weizmann.ac.il/LS_CoreFacilities/units/targeted-metabolomics/resources
",

"https://www.weizmann.ac.il/LS_CoreFacilities/targeted-metabolomics/sample-preparat
ion",

"https://www.weizmann.ac.il/LS_CoreFacilities/targeted-metabolomics/staff-and-conta
ct",

"https://www.weizmann.ac.il/LS_CoreFacilities/targeted-metabolomics/support",

"https://www.weizmann.ac.il/LS_CoreFacilities/targeted-metabolomics/safety",

```
"https://www.weizmann.ac.il/LS_CoreFacilities/targeted-metabolomics/education"
    ],
    "Viral Vector": [
        "https://www.weizmann.ac.il/LS_CoreFacilities/viral-vector/about",
        "https://www.weizmann.ac.il/LS_CoreFacilities/viral-vector/how-order",

"https://www.weizmann.ac.il/LS_CoreFacilities/viral-vector/sample-preparation",
        "https://www.weizmann.ac.il/LS_CoreFacilities/viral-vector/support",

"https://www.weizmann.ac.il/LS_CoreFacilities/viral-vector/staff-and-contacts"
    ],
    "Multidisciplinary Vesicle Program - MVP": [

"https://www.weizmann.ac.il/LS_CoreFacilities/multidisciplinary-vesicle-program-mvp
/about",

"https://www.weizmann.ac.il/LS_CoreFacilities/multidisciplinary-vesicle-program-mvp
/devices",

"https://www.weizmann.ac.il/LS_CoreFacilities/multidisciplinary-vesicle-program-mvp
/courses",

"https://www.weizmann.ac.il/LS_CoreFacilities/multidisciplinary-vesicle-program-mvp
/publications",

"https://www.weizmann.ac.il/LS_CoreFacilities/multidisciplinary-vesicle-program-mvp
/upcoming-events-click-registration",

"https://www.weizmann.ac.il/LS_CoreFacilities/multidisciplinary-vesicle-program-mvp
/staff-and-contacts",

"https://www.weizmann.ac.il/LS_CoreFacilities/multidisciplinary-vesicle-program-mvp
/how-order"
    ]
}

out_base = Path("OUTPUT_DIR")
out_base.mkdir(exist_ok=True)

for unit_name, urls in UNIT_URLS.items():
    print(f"Scraping {unit_name} ({len(urls)} pages)")

    # Request main-content markdown only
    batch = firecrawl.batch_scrape(
        urls,
        formats=["markdown"],          # only markdown [web:7][web:21]
        only_main_content=True,        # only main content (equivalent to
onlyMainContent) [web:7][web:17]
        max_age=172800000,             # optional cache age in ms (2 days)
[web:7][web:22]
```

```python
    )

    unit_folder = out_base / safe_filename(unit_name)
    unit_folder.mkdir(exist_ok=True)

    pages = getattr(batch, "data", []) or []

    # MASTER FILE: UnitName_FULL.md (main content markdown only)
    full_md = [f"# {unit_name.upper()}", f"**{len(pages)} MAIN-CONTENT PAGES**"]
    for i, doc in enumerate(pages):
        metadata = getattr(doc, "metadata", {})
        title = safe_filename(getattr(metadata, "title", f"Page_{i+1}"))
        source_url = getattr(metadata, "source_url", "")
        markdown = getattr(doc, "markdown", "") or ""

        full_md.extend(
            [
                "\n" + "=" * 80,
                f"## PAGE {i+1}: {title}",
                f"**URL:** {source_url}",
                "",
                markdown,
                "=" * 80,
            ]
        )

    md_file = unit_folder / f"{safe_filename(unit_name)}_FULL.md"
    md_file.write_text("\n".join(full_md), encoding="utf-8")

    # Individual pages (main-content markdown only)
    for i, doc in enumerate(pages):
        metadata = getattr(doc, "metadata", {})
        safe_title = safe_filename(getattr(metadata, "title", f"page_{i+1}"))
        page_dir = unit_folder / f"p{i+1}_{safe_title}"
        page_dir.mkdir(exist_ok=True)

        markdown = getattr(doc, "markdown", "") or ""
        (page_dir / "markdown_main.md").write_text(markdown, encoding="utf-8")

    print(f"{len(pages)} pages → {unit_folder}")

print(f"\nSAVED: {out_base}")
```
