# Supplementary material for: Improving core facility service discovery with an AI assistant grounded in institutional web content
Source: J Biomol Tech. 2026 Jun 27;37(2):40–9. doi: 10.7171/001c.162898 (PMC13313189; doi:10.7171/001c.162898)
Supplement: Supplemental File [file jbt_2026_37_2_162898_347720.pdf]

```
{
  "name": "Gemini File Search template",
  "nodes": [
    {
      "parameters": {
        "formTitle": "Doc Upload",
        "formFields": {
          "values": [
            {
              "fieldLabel": "File",
              "fieldType": "file",
              "requiredField": true
            }
          ]
        },
        "options": {}
      },
      "type": "n8n-nodes-base.formTrigger",
      "typeVersion": 2.3,
      "position": [
        672,
        400
      ],
      "id": "5eca4dfc-6c2b-4a49-b30a-1fc34b33877f",
      "name": "On form submission",
      "webhookId": "2fc51c8e-e886-4953-a527-7d9273db9508"
    },
    {
      "parameters": {},
      "type": "n8n-nodes-base.manualTrigger",
      "typeVersion": 1,
      "position": [
        736,
        96
      ],
      "id": "8d877c22-f077-47d4-a2be-370463276370",
      "name": "When clicking ‘Execute workflow’"
    },
    {
      "parameters": {
        "method": "POST",
        "url": "https://generativelanguage.googleapis.com/v1beta/fileSearchStores",
        "authentication": "genericCredentialType",
        "genericAuthType": "httpQueryAuth",
        "sendHeaders": true,
        "headerParameters": {
          "parameters": [
            {
              "name": "Content-Type",
              "value": "application/json"
```

```
          }
        ]
      },
      "sendBody": true,
      "bodyParameters": {
        "parameters": [
          {
            "name": "displayName",
            "value": "LSCF_GINCPM_chatbot"
          }
        ]
      },
      "options": {}
    },
    "type": "n8n-nodes-base.httpRequest",
    "typeVersion": 4.3,
    "position": [
      928,
      96
    ],
    "id": "d26cc933-19af-4091-8a64-6d78350605f1",
    "name": "Create File Store",
    "credentials": {
      "httpQueryAuth": {
        "id": "lBzGuBquNyQeIRb6",
        "name": "google_gemini_api"
      }
    }
  },
  {
    "parameters": {
      "method": "POST",
      "url": "=https://generativelanguage.googleapis.com/upload/v1beta/files",
      "authentication": "genericCredentialType",
      "genericAuthType": "httpQueryAuth",
      "sendBody": true,
      "contentType": "binaryData",
      "inputDataFieldName": "File",
      "options": {}
    },
    "type": "n8n-nodes-base.httpRequest",
    "typeVersion": 4.3,
    "position": [
      880,
      400
    ],
    "id": "d4e3106f-03ec-44e3-8e73-178078b667a1",
    "name": "Upload File",
    "credentials": {
      "httpQueryAuth": {
```

```
          "id": "lBzGuBquNyQeIRb6",
          "name": "google_gemini_api"
        }
      }
    },
    {
      "parameters": {
        "method": "POST",
        "url":
"=https://generativelanguage.googleapis.com/v1beta/fileSearchStores/fileSearchStore
s/lscfgincpmchatbot-c89xcdwoeaka:importFile",
        "authentication": "genericCredentialType",
        "genericAuthType": "httpQueryAuth",
        "sendHeaders": true,
        "headerParameters": {
          "parameters": [
            {
              "name": "Content-Type",
              "value": "application/json"
            }
          ]
        },
        "sendBody": true,
        "bodyParameters": {
          "parameters": [
            {
              "name": "file_name",
              "value": "={{ $json.file.name }}"
            }
          ]
        },
        "options": {}
      },
      "type": "n8n-nodes-base.httpRequest",
      "typeVersion": 4.3,
      "position": [
        1072,
        400
      ],
      "id": "5ec272e9-d3dd-4ef7-abda-58f9d9764aa4",
      "name": "Import File",
      "credentials": {
        "httpQueryAuth": {
          "id": "lBzGuBquNyQeIRb6",
          "name": "google_gemini_api"
        }
      }
    },
    {
      "parameters": {
```

```
      "content": "##  Create Store",
      "height": 288,
      "width": 704,
      "color": 6
    },
    "type": "n8n-nodes-base.stickyNote",
    "typeVersion": 1,
    "position": [
      592,
      0
    ],
    "id": "c917df28-f6e5-4904-a18d-19ac9800822a",
    "name": "Sticky Note"
  },
  {
    "parameters": {
      "content": "##  Upload File\n",
      "height": 288,
      "width": 704,
      "color": 6
    },
    "type": "n8n-nodes-base.stickyNote",
    "typeVersion": 1,
    "position": [
      592,
      304
    ],
    "id": "f101c9bc-eb4e-45cf-8f50-26c0b706dc8e",
    "name": "Sticky Note1"
  },
  {
    "parameters": {
      "options": {}
    },
    "type": "@n8n/n8n-nodes-langchain.chatTrigger",
    "typeVersion": 1.4,
    "position": [
      704,
      672
    ],
    "id": "c46c16f6-5542-43fa-9a75-e9ee445ce528",
    "name": "When chat message received",
    "webhookId": "f61815ce-f800-4913-a5d3-69303910022f"
  },
  {
    "parameters": {
      "content": "##  Query\n",
      "height": 288,
      "width": 704,
      "color": 5
```

```
      },
      "type": "n8n-nodes-base.stickyNote",
      "typeVersion": 1,
      "position": [
        592,
        608
      ],
      "id": "f0fcee10-a0ef-4aa0-bbf4-0c55416e230b",
      "name": "Sticky Note2"
    },
    {
      "parameters": {
        "method": "POST",
        "url":
"https://generativelanguage.googleapis.com/v1beta/models/gemini-2.5-flash:generateC
ontent",
        "authentication": "genericCredentialType",
        "genericAuthType": "httpQueryAuth",
        "sendBody": true,
        "specifyBody": "json",
        "jsonBody": "={\n  \"contents\": [\n    {\n      \"parts\": [\n        {
\"text\": \"{{ $fromAI(\"query\",\"the question the user needs an answer to\") }}\"
}\n      ]\n    }\n  ],\n  \"tools\": [\n    {\n      \"file_search\": {\n
\"file_search_store_names\": [\n
\"fileSearchStores/lscfgincpmchatbot-c89xcdwoeaka\"\n        ]\n      }\n    }\n
]\n}\n",
        "options": {}
      },
      "type": "n8n-nodes-base.httpRequestTool",
      "typeVersion": 4.3,
      "position": [
        1152,
        752
      ],
      "id": "6da77511-9c02-4986-a5a6-77091386d56a",
      "name": "Knowledge Base",
      "credentials": {
        "httpQueryAuth": {
          "id": "lBzGuBquNyQeIRb6",
          "name": "google_gemini_api"
        }
      }
    },
    {
      "parameters": {
        "options": {
          "systemMessage": "=You are a helpful RAG agent. Your job is to answer the
user's question using your Knowledge Base tool to make sure all of your answers are
grounded in truth. Please cite your sources when you're giving your answers.
\n\nWhen you are sending a query to the Knowledge Base tool, only send over text.
```

```
No punctuation, quotation marks, or new lines. "
        }
      },
      "type": "@n8n/n8n-nodes-langchain.agent",
      "typeVersion": 3,
      "position": [
        880,
        672
      ],
      "id": "c9dbc897-0988-4b3c-a517-b8e8b5947cbb",
      "name": "RAG Agent"
    },
    {
      "parameters": {
        "content": "# ⚙️ Setup Instructions\n\nFollow these steps to get your
Gemini-based file Q&A system up and running:\n\n### ☑️ Step 1: Obtain Your [Gemini
API Key](https://aistudio.google.com/app/apikey)  \nRequest your Gemini API key,
then insert it into the HTTP Request node — this is what enables your agent to
generate responses.\n\n### ☑️ Step 2: Connect Your File Store  \nConfirm that your
***file store name*** matches in both the `Import File` and `Knowledge Base` HTTP
Request nodes. This is essential for storing and retrieving your documents
accurately.\n\n### ☑️ Step 3: Upload Your Markdown/PDFs  \nUpload the PDFs you'd
like to use for testing.\n\n",
        "height": 432,
        "width": 576
      },
      "type": "n8n-nodes-base.stickyNote",
      "typeVersion": 1,
      "position": [
        0,
        0
      ],
      "id": "38c982d9-4b34-4d95-9cc9-71cb7d78f588",
      "name": "Sticky Note4"
    },
    {
      "parameters": {
        "options": {}
      },
      "type": "@n8n/n8n-nodes-langchain.lmChatGoogleGemini",
      "typeVersion": 1,
      "position": [
        832,
        832
      ],
      "id": "70bda85f-9339-40b2-afa6-7d5d1ba462f0",
      "name": "Google Gemini Chat Model",
      "credentials": {
        "googlePalmApi": {
          "id": "MeUn93nZR4bRThw7",
```

```
        "name": "gemini_api_key"
      }
    }
  }
],
"pinData": {
  "Create File Store": [
    {
      "json": {
        "name": "fileSearchStores/lscfgincpmchatbot-c89xcdwoeaka",
        "displayName": "LSCF_GINCPM_chatbot",
        "createTime": "2026-01-29T10:07:39.302971Z",
        "updateTime": "2026-01-29T10:07:39.302971Z"
      },
      "pairedItem": {
        "item": 0
      }
    }
  ]
},
"connections": {
  "On form submission": {
    "main": [
      [
        {
          "node": "Upload File",
          "type": "main",
          "index": 0
        }
      ]
    ]
  },
  "When clicking ‘Execute workflow’": {
    "main": [
      [
        {
          "node": "Create File Store",
          "type": "main",
          "index": 0
        }
      ]
    ]
  },
  "Upload File": {
    "main": [
      [
        {
          "node": "Import File",
          "type": "main",
          "index": 0
```

```
          }
        ]
      ]
    },
    "When chat message received": {
      "main": [
        [
          {
            "node": "RAG Agent",
            "type": "main",
            "index": 0
          }
        ]
      ]
    },
    "Knowledge Base": {
      "ai_tool": [
        [
          {
            "node": "RAG Agent",
            "type": "ai_tool",
            "index": 0
          }
        ]
      ]
    },
    "Google Gemini Chat Model": {
      "ai_languageModel": [
        [
          {
            "node": "RAG Agent",
            "type": "ai_languageModel",
            "index": 0
          }
        ]
      ]
    }
  },
  "active": false,
  "settings": {
    "executionOrder": "v1",
    "binaryMode": "separate",
    "availableInMCP": false
  },
  "versionId": "e5446109-30b7-4962-b85c-d5efcf4156ed",
  "meta": {
    "templateCredsSetupCompleted": true,
    "instanceId":
"291ec9456a92fea5d5976be252f308df9e94635b030bfa3fd5fe118344a5eead"
  },
```

"id": "W0vXC37ozmD7dPIG",
"tags": []
}
