# Supplementary material for: Improving core facility service discovery with an AI assistant grounded in institutional web content
Source: J Biomol Tech. 2026 Jun 27;37(2):40–9. doi: 10.7171/001c.162898 (PMC13313189; doi:10.7171/001c.162898)
Supplement: Supplemental File [file jbt_2026_37_2_162898_347721.pdf]

```python
# create_store.py
import argparse
from google import genai

def parse_args():
    parser = argparse.ArgumentParser(
        description="Create a Gemini File Search store."
    )
    parser.add_argument(
        "-name",
        "--name",
        required=True,
        help="Display name for the File Search store.",
    )
    parser.add_argument(
        "-api",
        "--api",
        required=True,
        help="Path to a text file containing the Gemini API key.",
    )
    return parser.parse_args()

def read_api_key(path: str) -> str:
    with open(path, "r", encoding="utf-8") as f:
        # strip to remove newline / spaces
        return f.read().strip()

def main():
    args = parse_args()
    display_name = args.name
    api_key = read_api_key(args.api)

    client = genai.Client(api_key=api_key)

    file_search_store = client.file_search_stores.create(
        config={"display_name": display_name}
    )  # Creates a new FileSearchStore.[web:6]

    print("Created File Search store:")
    print(f"  display_name: {display_name}")
    print(f"  name (use this in other scripts): {file_search_store.name}")

if __name__ == "__main__":
    main()
```
