# Supplementary material for: Improving core facility service discovery with an AI assistant grounded in institutional web content
Source: J Biomol Tech. 2026 Jun 27;37(2):40–9. doi: 10.7171/001c.162898 (PMC13313189; doi:10.7171/001c.162898)
Supplement: Supplemental File [file jbt_2026_37_2_162898_347722.pdf]

```python
# upload_dir_to_store.py
import argparse
import os
import time
from google import genai

def parse_args():
    parser = argparse.ArgumentParser(
        description="Upload all Markdown files in a directory to a Gemini File
Search store."
    )
    parser.add_argument(
        "-store",
        "--store",
        required=True,
        help="Full File Search store name (e.g. fileSearchStores/xxxx).",
    )
    parser.add_argument(
        "-path",
        "--path",
        required=True,
        help="Directory path containing .md files.",
    )
    parser.add_argument(
        "-api",
        "--api",
        required=True,
        help="Path to a text file containing the Gemini API key.",
    )
    return parser.parse_args()

def read_api_key(path: str) -> str:
    with open(path, "r", encoding="utf-8") as f:
        return f.read().strip()

def upload_file_to_store(client, store_name: str, filepath: str):
    print(f"Uploading: {filepath}")

    operation = client.file_search_stores.upload_to_file_search_store(
        file_search_store_name=store_name,
        file=filepath,  # direct upload into store[web:53][web:15]
        config={
            "display_name": os.path.basename(filepath),
            "chunking_config": {
                "white_space_config": {
                    "max_tokens_per_chunk": 200,
                    "max_overlap_tokens": 20,
                }
            },
        },
```

```
    )

    # Poll until done
    while not operation.done:
        print("  Waiting for operation to complete...")
        time.sleep(5)
        operation = client.operations.get(operation)

    if getattr(operation, "error", None):
        print(f"  Error for {filepath}: {operation.error}")
    else:
        print(f"  Done: {filepath}")

def main():
    args = parse_args()
    store_name = args.store
    directory = args.path
    api_key_path = args.api

    api_key = read_api_key(api_key_path)
    client = genai.Client(api_key=api_key)

    if not os.path.isdir(directory):
        raise SystemExit(f"Path is not a directory: {directory}")

    for entry in os.listdir(directory):
        full = os.path.join(directory, entry)
        if not os.path.isfile(full):
            continue
        if not entry.lower().endswith(".md"):
            continue
        upload_file_to_store(client, store_name, full)

if __name__ == "__main__":
    main()
```
