# Supplementary material for: Improving core facility service discovery with an AI assistant grounded in institutional web content
Source: J Biomol Tech. 2026 Jun 27;37(2):40–9. doi: 10.7171/001c.162898 (PMC13313189; doi:10.7171/001c.162898)
Supplement: Supplemental File [file jbt_2026_37_2_162898_347723.pdf]

```
{
  "name": "Gemini File Search",
  "nodes": [
    {
      "parameters": {
        "public": true,
        "options": {}
      },
      "type": "@n8n/n8n-nodes-langchain.chatTrigger",
      "typeVersion": 1.4,
      "position": [
        864,
        880
      ],
      "id": "18996957-6e14-44ca-92f6-bc174cededf7",
      "name": "When chat message received",
      "webhookId": "b47ef4c6-aaf8-4891-8fe9-f9cfb114dde9"
    },
    {
      "parameters": {
        "content": "##  Query\n",
        "height": 384,
        "width": 832,
        "color": 5
      },
      "type": "n8n-nodes-base.stickyNote",
      "typeVersion": 1,
      "position": [
        800,
        752
      ],
      "id": "78920b94-8e8c-421d-af94-c14f5ecc3d05",
      "name": "Sticky Note2"
    },
    {
      "parameters": {
        "method": "POST",
        "url":
"https://generativelanguage.googleapis.com/v1beta/models/gemini-2.5-flash:generateC
ontent",
        "authentication": "genericCredentialType",
        "genericAuthType": "httpQueryAuth",
        "sendBody": true,
        "specifyBody": "json",
        "jsonBody": "={\n  \"contents\": [\n    {\n      \"parts\": [\n        {
\"text\": \"{{ $fromAI(\"query\",\"the question the user needs an answer to\") }}\"
}\n      ]\n    }\n  ],\n  \"tools\": [\n    {\n      \"file_search\": {\n
\"file_search_store_names\": [\n
\"fileSearchStores/lscfgincpmchatbot-c89xcdwoeaka\"\n        ]\n      }\n    }\n
]\n}\n",
```

```
          "options": {}
        },
        "type": "n8n-nodes-base.httpRequestTool",
        "typeVersion": 4.3,
        "position": [
          1344,
          992
        ],
        "id": "297d6c0b-5bd9-4ec1-b2cb-dd37eb186723",
        "name": "Knowledge Base",
        "credentials": {
          "httpQueryAuth": {
            "id": "lBzGuBquNyQeIRb6",
            "name": "google_gemini_api"
          }
        }
      },
      {
        "parameters": {
          "options": {
            "systemMessage": "=You are the specialized Research Assistant for the
Life Sciences Core Facilities (LSCF) and the G-INCPM at the Weizmann Institute.
Your goal is to connect users to the exact resource they need with surgical
precision.\n\nOperational Protocol:\nTool Priority 1: Knowledge Base (KB). Search
here for any specific service, instrument, or Weizmann-specific policy.\nTool
Priority 2: Perplexity. Use this only for educational/theoretical context (e.g.,
\"How does CRISPR work?\") or when the KB confirms a service exists but lacks
technical depth.\nSynthesis: When combined, start with the Weizmann-specific
unit/link, then provide the scientific context from Perplexity.\n\nWhen you are
sending a query to the Knowledge Base tool, only send over text. No punctuation,
quotation marks, or new lines.\n\nThe \"Metabolomics Trinity\" & G-INCPM
Rules\nSmall Molecule Queries: Any mention of \"metabolites,\" \"lipids,\" or
\"small molecules\" must trigger a mention of all three: Metabolic Profiling,
Targeted Metabolomics, and Mass Spectrometry Imaging.\nSpatial Clause: If
\"imaging\" or \"localization\" is mentioned, highlight that Mass Spectrometry
Imaging is the only unit providing spatial maps.\n\nG-INCPM Projects: Only If a
G-INCPM unit is mentioned, include: [Submit a G-INCPM
Project].(https://g-incpm.weizmann.ac.il/eform/submit/project)\n\nResponse
Structure\nSummary: Briefly state which unit(s) handle the request.\nService
Details: Use KB data for specific instrumentation/ordering info.\nContext
(Optional): Use Perplexity for scientific background if the user asked \"why\" or
\"how.\"\nActionable Info: Contact details (specifically Genomics/Sandbox:
08-934-5168 / INCPM.samples@weizmann.ac.il) and mandatory links.\n\nOutput
formatting for links\nAlways present unit and web resources as Markdown hyperlinks
in the form [unit name](URL).\nExample: [Bioinformatics
G-INCPM](https://g-incpm.weizmann.ac.il/units/MantouxBioinformatics/about) and
[Submit a G-INCPM
Project](https://g-incpm.weizmann.ac.il/eform/submit/project).\nNever show raw URLs
unless the user explicitly asks for “plain URL”.\n\nFor every response that
mentions a specific unit, include a Markdown link using the unit name as the anchor
```

```
text, for example:\n[Genomics:
G-INCPM](https://g-incpm.weizmann.ac.il/units/CrownGenomics/about).\n\n"
        }
      },
      "type": "@n8n/n8n-nodes-langchain.agent",
      "typeVersion": 3,
      "position": [
        1216,
        768
      ],
      "id": "54cb2abb-e59c-4165-9948-de2ffbc40fce",
      "name": "RAG Agent"
    },
    {
      "parameters": {
        "options": {
          "temperature": 0.2
        }
      },
      "type": "@n8n/n8n-nodes-langchain.lmChatGoogleGemini",
      "typeVersion": 1,
      "position": [
        1088,
        992
      ],
      "id": "7f103fa5-4441-400a-bc26-622df75b6847",
      "name": "Google Gemini Chat Model",
      "credentials": {
        "googlePalmApi": {
          "id": "MeUn93nZR4bRThw7",
          "name": "gemini_api_key"
        }
      }
    },
    {
      "parameters": {
        "messages": {
          "message": [
            {
              "content": "You are a helpful assistant for GINCPM and LSCF units.
Answer using only provided context/search results from
https://www.weizmann.ac.il/LS_CoreFacilities/,
https://g-incpm.weizmann.ac.il/.\n\nRespond in neutral expert tone with ###/####
headers, short paras/lists, tables for comparisons. Explicitly state \"no
information found\" if missing.\n\nFor units: Always append [Unit Name](source
URL). G-INCPM: + [Start a
project](https://g-incpm.weizmann.ac.il/eform/submit/project).\n\nG-INCPM Units
(6): Bioinformatics: G-INCPM
[https://g-incpm.weizmann.ac.il/units/MantouxBioinformatics/about](https://g-incpm.
weizmann.ac.il/units/MantouxBioinformatics/about), Drug Discovery: G-INCPM
```

https://g-incpm.weizmann.ac.il/units/WohlDrugDiscovery/about, Genomics: G-INCPM https://g-incpm.weizmann.ac.il/units/CrownGenomics/about, Medicinal Chemistry: G-INCPM https://g-incpm.weizmann.ac.il/units/MedicinalChemistry/about, Protein Profiling: G-INCPM https://g-incpm.weizmann.ac.il/units/deBottonProteinProfiling/about, Information Technology: G-UNCPM https://g-incpm.weizmann.ac.il/units/InformationTechnology/about.\n\nLSCF Units (24): Antibody Engineering https://www.weizmann.ac.il/LS_CoreFacilities/antibody/about, Bacteriology & Genomic repository https://www.weizmann.ac.il/LS_CoreFacilities/bacteriology-genomic-repository/about, Bioinformatics: LSCF https://www.weizmann.ac.il/LS_CoreFacilities/bioinformatics-lscf/about, Biomedical Research https://www.weizmann.ac.il/LS_CoreFacilities/biomedical-research/about, Crystallization, Structural Determination and Structure Modeling https://www.weizmann.ac.il/LS_CoreFacilities/Crystallisation-and-Structure-Determination/about, DNA Manipulation https://www.weizmann.ac.il/LS_CoreFacilities/dna-manipulation/about, DNA sequencing https://www.weizmann.ac.il/LS_CoreFacilities/dna-sequencing/about, Ecosystem Microbiome Research https://www.weizmann.ac.il/LS_CoreFacilities/ecosystem-microbiome-research/about, Flow Cytometry https://www.weizmann.ac.il/LS_CoreFacilities/flow-cytometry/about, Fly food https://www.weizmann.ac.il/LS_CoreFacilities/fly-food/about, Genomics, Sandbox https://www.weizmann.ac.il/LS_CoreFacilities/genomics-sandbox/about, Irradiation https://www.weizmann.ac.il/LS_CoreFacilities/irradiation/about, Mass Cytometry https://www.weizmann.ac.il/LS_CoreFacilities/mass-cytometry/about, Mass spectrometry imaging https://www.weizmann.ac.il/LS_CoreFacilities/mass-spectrometry-imaging/about, Metabolic Profiling https://www.weizmann.ac.il/LS_CoreFacilities/metabolic-profiling/about, MICC Cell Observatory https://www.weizmann.ac.il/LS_CoreFacilities/micc-cell-observatory/about, MRI

https://www.weizmann.ac.il/LS_CoreFacilities/mri/about, Multidisciplinary Vesicle Program (MVP) https://www.weizmann.ac.il/LS_CoreFacilities/multidisciplinary-vesicle-program-mvp/about, Protein Analysis https://www.weizmann.ac.il/LS_CoreFacilities/protein-analysis/about, Protein Expression https://www.weizmann.ac.il/LS_CoreFacilities/protein-expression/about, Protein Purification https://www.weizmann.ac.il/LS_CoreFacilities/protein-purification/about, Stem Cells & Organoids https://www.weizmann.ac.il/LS_CoreFacilities/stem-cells-organoids/about, Targeted Metabolomics https://www.weizmann.ac.il/LS_CoreFacilities/targeted-metabolomics/about, Viral Vector https://www.weizmann.ac.il/LS_CoreFacilities/viral-vector/about.\n\n\nHead of units: Antibody Engineering Unit - Dr. Ira Zaretsky ira.zaretsky@weizmann.ac.il +972-8-934-6741; Bacteriology & Genomic repository - Dr. Ghil Jona ghil.jona@weizmann.ac.il +972-8-934-6026; Ilana and Pascal Mantoux Institute for Bioinformatics - Dr. Dan Ben-Avraham dan.ben-avraham@weizmann.ac.il +972-8-934-6718; Bioinformatics: LSCF - Dr. Dena Leshkowitz dena.leshkowitz@weizmann.ac.il +972-8-934-6330; Biomedical Research - Aharon Peretz aharon.peretz@weizmann.ac.il +972-8-934-2258; Crystallization, Structural Determination and Structure Modeling - Dr. Orly Dym orly.dym@weizmann.ac.il +972-8-934-6030; DNA Manipulation - Dr. Yoav Peleg yoav.peleg@weizmann.ac.il +972-8-934-3191, Dr. Tamar Unger tamar.unger@weizmann.ac.il +972-8-934-2242; Maurice and Vivienne Wohl Institute for Drug Discovery - Dr. Haim Michael Barr haim.barr@weizmann.ac.il +972-8-934-4379; DNA sequencing - Shani Mor Karasenti shani-mor.karasenti@weizmann.ac.il +972-8-934-3688, Dalit Merhav dalit.merhav@weizmann.ac.il +972-8-934-4249; Ecosystem Microbiome Research - Dr. Dagan Sade dagan.sade@weizmann.ac.il +972-8-934-4403; Flow Cytometry - Dr. Ziv Porat ziv.porat@weizmann.ac.il +972-8-934-2235; Fly food - Shari Carmon shari.carmon@weizmann.ac.il +972-54-549-9889; Crown Institute for Genomics - Dr. Hadas Keren-Shaul INCPM.samples@weizmann.ac.il 08-9345168; Genomics, Sandbox - Dr. Hadas Keren-Shaul INCPM.samples@weizmann.ac.il 08-9345168; Information Technology (G-INCPM) - Sharon Dahan sharon.dahan@weizmann.ac.il +972-8-934-2658; Irradiation - Hedva Hamawi hedva.hamawi@weizmann.ac.il +972-8-934-2374; MICC Cell Observatory - Dr. Yoseph Addadi yoseph.addadi@weizmann.ac.il +972-8-934-6332; MRI - Dr. Edna Furman-Haran edna.haran@weizmann.ac.il +972-8-934-6098; Mass Cytometry - Dr. Tomer-Meir Salame tomer-meir.salame@weizmann.ac.il +972-8-934-4385; Mass spectrometry imaging - Dr. Herbert Uwe Heinig uwe.heinig@weizmann.ac.il +972-8-934-2774; Medicinal Chemistry (G-INCPM) - Dr. Haim Michael Barr haim.barr@weizmann.ac.il +972-8-934-4379; Metabolic Profiling - Dr. Sergey Malitsky sergey.malitsky@weizmann.ac.il +972-8-934-3551; Multidisciplinary Vesicle Program (MVP) - Dr. Avraham Dayan avraham.dayan@weizmann.ac.il (no phone); Protein Analysis - Dr. Yael Fridmann Sirkis yael.fridmann-sirkis@weizmann.ac.il +972-8-934-4340; Protein Expression - Dr. Tamar Unger tamar.unger@weizmann.ac.il +972-8-934-2242, Dr. Yoav Peleg

yoav.peleg@weizmann.ac.il +972-8-934-3191; de Botton Institute for Protein Profiling - Dr. Yishai Levin yishai.levin@weizmann.ac.il +972-8-934-4315; Protein Purification - Dr. Shira Albeck shira.albeck@weizmann.ac.il +972-8-934-4290; Stem Cells & Organoids - Dr. Elena Ainbinder elena.ainbinder@weizmann.ac.il +972-8-934-6539; Targeted Metabolomics - Dr. Alexander Brandis alexander.brandis@weizmann.ac.il +972-8-934-6532; Viral Vector - Dr. Oded Singer oded.singer@weizmann.ac.il +972-8-934-2159..\n",
```
              "role": "system"
            },
            {
              "content": "={{ /*n8n-auto-generated-fromAI-override*/
$fromAI('message1_Text', ``, 'string') }}"
            }
          ]
        },
        "options": {
          "temperature": "=0.2",
          "topK": 30,
          "searchDomainFilter":
"https://www.weizmann.ac.il/LS_CoreFacilities/,https://g-incpm.weizmann.ac.il/",
          "searchRecency": "month"
        },
        "requestOptions": {}
      },
      "id": "ec43dc0b-99b4-4445-b8ab-a360f23a42a9",
      "name": "Perplexity Tool",
      "type": "n8n-nodes-base.perplexityTool",
      "typeVersion": 1,
      "position": [
        1472,
        992
      ],
      "credentials": {
        "perplexityApi": {
          "id": "xEs0htTMLNgQI48l",
          "name": "Perplexity account Weizmann"
        }
      }
    },
    {
      "parameters": {
        "contextWindowLength": 3
      },
      "type": "@n8n/n8n-nodes-langchain.memoryBufferWindow",
      "typeVersion": 1.3,
      "position": [
        1216,
        992
      ],
      "id": "1ed9abd8-b70b-4985-a079-f4325e180601",
```

```
      "name": "Simple Memory"
    },
    {
      "parameters": {
        "documentId": {
          "__rl": true,
          "value": "1VPNmsUvDu-Ej5CEieFWO4B3TIYkCOj7LvO8fn-AWKOY",
          "mode": "list",
          "cachedResultName": "Gemini powered chatbot test questions",
          "cachedResultUrl":
"https://docs.google.com/spreadsheets/d/1VPNmsUvDu-Ej5CEieFWO4B3TIYkCOj7LvO8fn-AWKO
Y/edit?usp=drivesdk"
        },
        "sheetName": {
          "__rl": true,
          "value": "gid=0",
          "mode": "list",
          "cachedResultName": "Test Questions",
          "cachedResultUrl":
"https://docs.google.com/spreadsheets/d/1VPNmsUvDu-Ej5CEieFWO4B3TIYkCOj7LvO8fn-AWKO
Y/edit#gid=0"
        },
        "options": {}
      },
      "id": "22830814-7b58-4a7e-b2d9-c8224912dcdc",
      "name": "Read Test Questions",
      "type": "n8n-nodes-base.googleSheets",
      "typeVersion": 4.7,
      "position": [
        1088,
        1312
      ],
      "credentials": {
        "googleSheetsOAuth2Api": {
          "id": "MM8Cvbsn7mq3gHJk",
          "name": "Google Sheets account"
        }
      }
    },
    {
      "parameters": {
        "assignments": {
          "assignments": [
            {
              "id": "id-1",
              "name": "chatInput",
              "value": "={{ $json.question }}",
              "type": "string"
            },
            {
```

```
              "id": "id-2",
              "name": "sessionId",
              "value": "={{ 'test-' + $json.row_number }}",
              "type": "string"
            }
          ]
        },
        "includeOtherFields": true,
        "options": {}
      },
      "id": "217148fc-9dd7-476d-8d2c-03bad44e9f69",
      "name": "Format Test Question",
      "type": "n8n-nodes-base.set",
      "typeVersion": 3.4,
      "position": [
        1312,
        1312
      ]
    },
    {
      "parameters": {
        "promptType": "define",
        "text": "={{ $json.chatInput }}",
        "options": {
          "systemMessage": "You are the specialized Research Assistant for the Life
Sciences Core Facilities (LSCF) and the G-INCPM at the Weizmann Institute. Your
goal is to connect users to the exact resource they need with surgical
precision.\n\nOperational Protocol:\nTool Priority 1: Knowledge Base (KB). Search
here for any specific service, instrument, or Weizmann-specific policy.\nTool
Priority 2: Perplexity. Use this only for educational/theoretical context (e.g.,
\"How does CRISPR work?\") or when the KB confirms a service exists but lacks
technical depth.\nSynthesis: When combined, start with the Weizmann-specific
unit/link, then provide the scientific context from Perplexity.\n\nWhen you are
sending a query to the Knowledge Base tool, only send over text. No punctuation,
quotation marks, or new lines.\n\nThe \"Metabolomics Trinity\" & G-INCPM
Rules\nSmall Molecule Queries: Any mention of \"metabolites,\" \"lipids,\" or
\"small molecules\" must trigger a mention of all three: Metabolic Profiling,
Targeted Metabolomics, and Mass Spectrometry Imaging.\nSpatial Clause: If
\"imaging\" or \"localization\" is mentioned, highlight that Mass Spectrometry
Imaging is the only unit providing spatial maps.\n\nG-INCPM Projects: If a G-INCPM
unit is mentioned, you must include: [Submit a G-INCPM
Project].(https://g-incpm.weizmann.ac.il/eform/submit/project)\n\nResponse
Structure\nSummary: Briefly state which unit(s) handle the request.\nService
Details: Use KB data for specific instrumentation/ordering info.\nContext
(Optional): Use Perplexity for scientific background if the user asked \"why\" or
\"how.\"\nActionable Info: Contact details (specifically Genomics/Sandbox:
08-934-5168 / INCPM.samples@weizmann.ac.il) and mandatory links.\n\nOutput
formatting for links\nAlways present unit and web resources as Markdown hyperlinks
in the form [unit name](URL).\nExample: [Bioinformatics
G-INCPM](https://g-incpm.weizmann.ac.il/units/MantouxBioinformatics/about) and
```

```
[Submit a G-INCPM
Project](https://g-incpm.weizmann.ac.il/eform/submit/project).\nNever show raw URLs
unless the user explicitly asks for “plain URL”.\n\nFor every response that
mentions a specific unit, include a Markdown link using the unit name as the anchor
text, for example:\n[Genomics:
G-INCPM](https://g-incpm.weizmann.ac.il/units/CrownGenomics/about)."
        }
      },
      "id": "dd7015ba-d887-4560-9eeb-94e05a3d7af0",
      "name": "Test AI Agent",
      "type": "@n8n/n8n-nodes-langchain.agent",
      "typeVersion": 2.2,
      "position": [
        1600,
        1312
      ]
    },
    {
      "parameters": {
        "assignments": {
          "assignments": [
            {
              "id": "id-1",
              "name": "timestamp",
              "value": "={{ $now.toISO() }}",
              "type": "string"
            },
            {
              "id": "id-2",
              "name": "test_question",
              "value": "={{ $('Format Test Question').item.json.chatInput }}",
              "type": "string"
            },
            {
              "id": "id-3",
              "name": "agent_response",
              "value": "={{ $json.output }}",
              "type": "string"
            },
            {
              "id": "id-4",
              "name": "test_id",
              "value": "={{ $('Format Test Question').item.json.sessionId }}",
              "type": "string"
            }
          ]
        },
        "includeOtherFields": true,
        "options": {}
      },
```

```
      "id": "2e52bf5a-da21-480a-a4c8-7becbb544f5b",
      "name": "Prepare Test Results",
      "type": "n8n-nodes-base.set",
      "typeVersion": 3.4,
      "position": [
        2000,
        1312
      ]
    },
    {
      "parameters": {
        "operation": "append",
        "documentId": {
          "__rl": true,
          "value": "1VPNmsUvDu-Ej5CEieFWO4B3TIYkCOj7LvO8fn-AWKOY",
          "mode": "list",
          "cachedResultName": "Gemini powered chatbot test questions",
          "cachedResultUrl":
"https://docs.google.com/spreadsheets/d/1VPNmsUvDu-Ej5CEieFWO4B3TIYkCOj7LvO8fn-AWKO
Y/edit?usp=drivesdk"
        },
        "sheetName": {
          "__rl": true,
          "value": 742970865,
          "mode": "list",
          "cachedResultName": "Stress Test Prompts",
          "cachedResultUrl":
"https://docs.google.com/spreadsheets/d/1VPNmsUvDu-Ej5CEieFWO4B3TIYkCOj7LvO8fn-AWKO
Y/edit#gid=742970865"
        },
        "columns": {
          "mappingMode": "defineBelow",
          "value": {
            "agent_response": "={{ $json.agent_response }}",
            "test_question": "={{ $json.test_question }}",
            "test_id": "={{ $json.test_id }}",
            "timestamp": "={{ $json.timestamp }}"
          },
          "matchingColumns": [],
          "schema": [
            {
              "id": "timestamp",
              "displayName": "timestamp",
              "required": false,
              "defaultMatch": false,
              "display": true,
              "type": "string",
              "canBeUsedToMatch": true
            },
            {
```

```
    "id": "test_id",
    "displayName": "test_id",
    "required": false,
    "defaultMatch": false,
    "display": true,
    "type": "string",
    "canBeUsedToMatch": true
  },
  {
    "id": "test_question",
    "displayName": "test_question",
    "required": false,
    "defaultMatch": false,
    "display": true,
    "type": "string",
    "canBeUsedToMatch": true
  },
  {
    "id": "agent_response",
    "displayName": "agent_response",
    "required": false,
    "defaultMatch": false,
    "display": true,
    "type": "string",
    "canBeUsedToMatch": true
  },
  {
    "id": "Models",
    "displayName": "Models",
    "required": false,
    "defaultMatch": false,
    "display": true,
    "type": "string",
    "canBeUsedToMatch": true,
    "removed": false
  },
  {
    "id": "Correctness and factual grounding",
    "displayName": "Correctness and factual grounding",
    "required": false,
    "defaultMatch": false,
    "display": true,
    "type": "string",
    "canBeUsedToMatch": true,
    "removed": false
  },
  {
    "id": "Relevance and routing to cores/contacts",
    "displayName": "Relevance and routing to cores/contacts",
    "required": false,
```

```
          "defaultMatch": false,
          "display": true,
          "type": "string",
          "canBeUsedToMatch": true,
          "removed": false
        },
        {
          "id": "Completeness and usefulness",
          "displayName": "Completeness and usefulness",
          "required": false,
          "defaultMatch": false,
          "display": true,
          "type": "string",
          "canBeUsedToMatch": true,
          "removed": false
        },
        {
          "id": "Style and clarity",
          "displayName": "Style and clarity",
          "required": false,
          "defaultMatch": false,
          "display": true,
          "type": "string",
          "canBeUsedToMatch": true,
          "removed": false
        }
      ],
      "attemptToConvertTypes": false,
      "convertFieldsToString": false
    },
    "options": {}
  },
  "id": "47de516b-41ec-40e8-87e2-312015f79bc5",
  "name": "Write Test Results",
  "type": "n8n-nodes-base.googleSheets",
  "typeVersion": 4.7,
  "position": [
    2224,
    1312
  ],
  "credentials": {
    "googleSheetsOAuth2Api": {
      "id": "MM8Cvbsn7mq3gHJk",
      "name": "Google Sheets account"
    }
  }
},
{
  "parameters": {},
  "type": "n8n-nodes-base.manualTrigger",
```

```
      "typeVersion": 1,
      "position": [
        864,
        1312
      ],
      "id": "b8e60c15-67e9-41ec-a8d1-b0f00708f08b",
      "name": "Execute workflow"
    },
    {
      "parameters": {
        "content": "##  Test Models",
        "height": 448,
        "width": 1616,
        "color": 4
      },
      "type": "n8n-nodes-base.stickyNote",
      "typeVersion": 1,
      "position": [
        832,
        1232
      ],
      "id": "05059f25-82d9-4094-bd67-77ebd0cef61f",
      "name": "Sticky Note3"
    }
  ],
  "pinData": {},
  "connections": {
    "When chat message received": {
      "main": [
        [
          {
            "node": "RAG Agent",
            "type": "main",
            "index": 0
          }
        ]
      ]
    },
    "Knowledge Base": {
      "ai_tool": [
        [
          {
            "node": "RAG Agent",
            "type": "ai_tool",
            "index": 0
          },
          {
            "node": "Test AI Agent",
            "type": "ai_tool",
            "index": 0
```

```
      }
    ]
  ]
},
"Google Gemini Chat Model": {
  "ai_languageModel": [
    [
      {
        "node": "RAG Agent",
        "type": "ai_languageModel",
        "index": 0
      },
      {
        "node": "Test AI Agent",
        "type": "ai_languageModel",
        "index": 0
      }
    ]
  ]
},
"Perplexity Tool": {
  "ai_tool": [
    [
      {
        "node": "RAG Agent",
        "type": "ai_tool",
        "index": 0
      },
      {
        "node": "Test AI Agent",
        "type": "ai_tool",
        "index": 0
      }
    ]
  ]
},
"Simple Memory": {
  "ai_memory": [
    [
      {
        "node": "RAG Agent",
        "type": "ai_memory",
        "index": 0
      }
    ]
  ]
},
"Read Test Questions": {
  "main": [
    [
```

```
          {
            "node": "Format Test Question",
            "type": "main",
            "index": 0
          }
        ]
      ]
    },
    "Format Test Question": {
      "main": [
        [
          {
            "node": "Test AI Agent",
            "type": "main",
            "index": 0
          }
        ]
      ]
    },
    "Test AI Agent": {
      "main": [
        [
          {
            "node": "Prepare Test Results",
            "type": "main",
            "index": 0
          }
        ]
      ]
    },
    "Prepare Test Results": {
      "main": [
        [
          {
            "node": "Write Test Results",
            "type": "main",
            "index": 0
          }
        ]
      ]
    },
    "Execute workflow": {
      "main": [
        [
          {
            "node": "Read Test Questions",
            "type": "main",
            "index": 0
          }
        ]
```

```
      ]
    }
  },
  "active": false,
  "settings": {
    "executionOrder": "v1",
    "availableInMCP": false
  },
  "versionId": "f4e75a5f-62f0-493f-a3a9-dc904cb4ab11",
  "meta": {
    "templateCredsSetupCompleted": true,
    "instanceId":
"291ec9456a92fea5d5976be252f308df9e94635b030bfa3fd5fe118344a5eead"
  },
  "id": "POWGXPYFEuBaPTfL",
  "tags": []
}
```
