# Supplementary material for: Improving core facility service discovery with an AI assistant grounded in institutional web content
Source: J Biomol Tech. 2026 Jun 27;37(2):40–9. doi: 10.7171/001c.162898 (PMC13313189; doi:10.7171/001c.162898)
Supplement: Supplemental File [file jbt_2026_37_2_162898_347724.pdf]

Note that actual links and contact names have been deleted in the following chats.

Q1:

Question: My antibody is weak how can it be improved:

Chat: If your antibody is "weak," it likely means you're looking to improve its binding affinity or overall effectiveness. The Antibody Engineering Unit can help you enhance your antibody through several specialized techniques:

- **Antibody Humanization:** This process involves engineering non-human antibodies to resemble human germline sequences. Beyond improving human compatibility, this approach can also **optimize antigen affinity** through structure-guided design, making your antibody bind more strongly to its target.
- **Novel Antibody Production through Single-Cell Sequencing:** This advanced method allows for the selection of B cells based on their antigen binding. By sequencing their antibody genes and then synthesizing and expressing these genes, the unit can **accelerate the generation of high-affinity antibodies**. This is particularly useful for discovering novel antibodies with desired specificities and improved binding characteristics.
- **Antibody-Protein Fusions:** While not directly increasing binding affinity, fusing your antibody with another functional protein (e.g., a cytokine or enzyme) can significantly **improve its overall effectiveness** by adding new functionalities, extending its half-life, or enhancing targeted delivery.
- **Bispecific Antibodies:** Engineering your antibody to bind two different antigens simultaneously can **enhance its efficacy** by enabling unique therapeutic mechanisms, such as redirecting immune cells or blocking multiple signaling pathways, even if the individual binding strength to a single target isn't dramatically increased.

The unit emphasizes a personalized approach, starting with a **personal consultation** to understand your specific needs and design a customized project plan to improve your antibody's performance.

For more details on how to improve your antibody, please contact the Antibody Engineering Unit.

Q2:

Question: how do I measure the affinity of my antibody:

Chat: To measure the affinity of your antibody, you'll need to use biophysical techniques that assess biomolecular interactions. The Protein Analysis Unit offers several "gold standard" methods for this.

- **Isothermal Titration Microcalorimetry (ITC) using ITC PEAQ:** This is considered a "gold standard" for studying biomolecule interactions. ITC directly measures the heat changes that occur when molecules bind, providing a label-free measurement of binding affinity and thermodynamics in a single experiment. It allows for the accurate determination of:
    - **Binding constants (KD)**: A direct measure of affinity.
    - **Reaction stoichiometry (N)**
    - **Enthalpy (ΔH)**
    - **Entropy (ΔS)** This provides a complete thermodynamic profile of the molecular interaction.
- **Microscale Thermophoresis (MST) using MST Monolith:** MST is a biophysical assay that quantifies interactions between molecules in solution. It detects changes in the movement of fluorescent molecules within a microscopic temperature gradient, which indicate alterations in the molecular hydration shell, charge, or size upon binding. The unit has various MST instruments, including Micro-, Red, Label-free, and Pico (Red and Blue) versions.
- **Surface Plasmon Resonance (SPR) using Biacore™ 8K:** This is a high-throughput, high-sensitivity SPR system used for efficient screening, characterization, process optimization, and quality control of biomolecule binding. The Biacore™ 8K system delivers high-quality binding data with high capacity.

While other techniques like Differential Scanning Fluorimetry (DSF) and Differential Scanning Calorimetry (DSC) are available, their primary focus is on thermal stability and unfolding, rather than direct measurement of binding affinity.

For detailed information and to discuss which method is best suited for your antibody, please contact the Protein Analysis Unit. You can also submit….

Q3:

Question: Measuring RNA expression

Chat: Measuring RNA expression is a fundamental process for understanding gene function and cellular mechanisms. The Genomics core offers several technologies for this purpose:

Technologies for RNA Expression Measurement:

- **NanoString:** This is a probe-based technology suitable for measuring the expression of several hundred genes per sample. It's particularly useful for samples that are not ideal for traditional RNA sequencing, such as FFPE (formalin-fixed paraffin-embedded) samples. NanoString offers specific panels for both gene expression and microRNA (miRNA) expression analysis.
- **RNA Sequencing (RNA-seq) Methods:** The unit also utilizes various RNA-seq methods, including:
    - Bulk MARS-seq
    - Illumina TruSeq Stranded mRNA
    - Bulk SMART-seq 2
    - FFPE bulk RNA-seq

Critical RNA Sample Quality Parameters:

To ensure accurate results, your RNA samples must meet specific quality criteria:

- **Concentration:**

    - Measured by Nanodrop or Qubit (Qubit is recommended for concentrations below ~40ng/µl for higher accuracy).
    - For NanoString gene expression panels: 10µl at 30ng/µl.
    - For NanoString miRNA panels: 6µl at 33ng/µl.
- **Purity Ratios:**
    - OD260/280 ratio should be ≥ 1.8 (or >1.9 for NanoString).
    - OD260/230 ratio should be ≥ 2 (or >1.8 for NanoString).
- **Integrity:**
    - Assessed using instruments like a TapeStation or Bioanalyzer to determine the RNA Integrity Number (RIN), which should ideally be ≥ 8.
    - For FFPE bulk RNA sequencing, a DV200 (percentage of RNA fragments ≥ 200 nucleotides) of >30% is recommended.
    - For NanoString, a DV200 of 50% and up is highly recommended.
    - *Note:* RIN numbers are based on human, mouse, and rat RNA profiles; other organisms may not generate high RIN numbers but can still be high quality. RNA samples that have undergone polyA selection or ribosomal depletion cannot be evaluated for RIN score.

For detailed information on sample submission and to discuss the best approach for your RNA expression experiments, please contact…

Q4:

Question: what methods can i use to sort cells

Chat: The primary method for cell sorting available at the Weizmann Institute is **Fluorescence-Activated Cell Sorting (FACS)**, offered by the Flow Cytometry Unit. This is a specialized type of flow cytometry that allows you to separate a heterogeneous population of cells into distinct subpopulations based on specific characteristics.

Here's how cell sorting works and the key considerations:

How Cell Sorting Works (FACS Principles):

1. **Sample Preparation:**
   - Cells are suspended in a single-cell solution, typically in Calcium and Magnesium-free PBS, with serum sometimes added.
   - **Crucially, all samples must be filtered through a nylon mesh (e.g., 40-50 µm) before being introduced to the flow cytometer.** This prevents clogging of the instrument's fluidics by clumps or debris.
2. **Cell Staining/Labeling:**
   - Cells are usually labeled with fluorescent markers (e.g., antibodies conjugated with fluorochromes) that bind to specific cell surface or intracellular components.
   - Light scatter properties (forward scatter for size, side scatter for granularity) also help in identifying cell populations.
   - Advanced instruments like the **Cytek™ Aurora** can capture the full emission spectrum, allowing characterization of up to 40 colors at a single-cell level.
3. **Hydrodynamic Focusing:**
   - The cell sample is introduced into a fluid stream that narrows, causing cells to pass individually in a single file line through one or more laser beams.
4. **Detection:**
   - As each cell passes through the laser, fluorescent markers are excited, emitting light at specific wavelengths.
   - Detectors capture this emitted light, along with forward and side scatter, to characterize each cell based on its unique fluorescent profile and physical properties.
5. **Droplet Formation and Sorting:**
   - Based on pre-defined criteria, the instrument decides whether to sort a particular cell.
   - The stream containing the cells is vibrated at a high frequency, breaking it into individual droplets.
   - If a droplet contains a target cell, it is given an electrical charge (positive or negative).
6. **Deflection:**

  - The charged droplets then pass through an electrostatic field, which deflects them into collection tubes or multi-well plates based on their charge. Uncharged droplets (containing unwanted cells or no cells) are discarded.

Q5:

Question: what sorters ar available

Chat: The Flow Cytometry Unit offers:

- **Conventional Cell Sorters:** These can analyze and sort particles ranging in size from 0.5 to 40 µm, precisely depositing selected populations into tubes or plates, often under sterile conditions. Instruments include:
  - 2 FACSAriaIII with 5 lasers
  - SORP-FACSAriaII with 5 lasers
  - FACSymphony S6 with 5 lasers
  - FACSAriaIII with 4 lasers
  - FACSAria FUSION with 4 lasers
- **Large Particle Sorter (BioSorter):** This instrument is designed for larger and/or more fragile cells, capable of analyzing and sorting objects ranging in size from 10 to 1,500 µm. It has 4 lasers and can trace fluorescence intensity changes across the entire length of the object.

Key Considerations for Cell Sorting:

- **Sterility:** Sterile sorting is often required, necessitating the use of sterile PBS and water, and specific machine preparation.
- **Nozzle Size:** The choice of nozzle size (e.g., 70 µm for high speed, 85 µm for medium, 100 or 130 µm for low speed) impacts sorting speed and the viability of delicate cells.
- **Speed:** Sorting speed can be adjusted, with higher speeds generally associated with smaller nozzle sizes.
- **Sample Media:** Cells to be sorted are suspended in Calcium and Magnesium-free PBS. Sorting media also consists of Calcium and Magnesium-free PBS, which must be sterile

for experiments requiring culture or functional analyses of sorted cells. Sorted cells are typically collected into tubes containing pure serum.

The Flow Cytometry Unit provides assistance during planning, execution, and analysis of experiments, with an emphasis on teaching users to become independent.

Q6.

Question: What sort of metabolites can be found in plants

Chat: You can measure a wide variety of metabolites in plant tissue using the Metabolic Profiling, Targeted Metabolomics, and Mass Spectrometry Imaging units.

Service Details:

These units utilize advanced mass spectrometry techniques to identify and quantify a broad range of compounds, providing insights into the metabolic state and spatial distribution of molecules within plant tissues.

- **Metabolic Profiling** and **Targeted Metabolomics** can identify and quantify:
  - Sugars
  - Organic acids
  - Amino acids
  - Phospholipids
  - Sphingolipids
  - Triacylglycerols
  - Pigments
  - Flavonols
  - Other specialized metabolites

  These analyses are performed using platforms that include UPLC and HPLC separation technologies coupled with various Mass Spectrometry detectors such as Q-Exactive, QTOF, LTQ XL Orbitrap, and LC-Triple Quad Mass Spectrometers.

- **Mass Spectrometry Imaging (MSI)** is the only unit that provides spatial maps, allowing for the direct visualization of hundreds of different molecules across a tissue sample. This creates an ion density map showing the precise location and distribution of metabolites within plant cells, tissues, and organs. MSI can detect both endogenous biomolecules (like sugars, organic acids, and lipids) and exogenous molecules. Key MSI techniques include:
  - **MALDI Imaging:** Offers spatial resolution typically ranging from 5 to 100 µm and can be coupled with high-resolution mass spectrometers.
  - **SIMS Imaging:** Provides very high lateral resolution (less than 100 nm) and is a matrix-free technology allowing for 3D acquisition.
  - **Orbi-SIMS Technology:** Combines an Argon gas cluster source with an Orbitrap™ mass analyzer for ultra-high mass resolution and excellent lateral resolution.

For further inquiries or to order services, please contact..

Q7

Question: which core facilities support extracellular vesicle research and why

Chat:The Multidisciplinary Vesicle Program (MVP) is the primary core facility supporting extracellular vesicle (EV) research at the Weizmann Institute.

Why the MVP and other core facilities support EV research:

Extracellular vesicles (EVs) are nanoscale particles crucial for intercellular communication, transferring proteins, lipids, and nucleic acids between cells. Their natural origin provides excellent biocompatibility and targeting capabilities, making them valuable for molecular biology, diagnostics, and gene therapy. EV research is inherently multidisciplinary, requiring a wide array of advanced instrumentation and specialized techniques, which are provided by the MVP in collaboration with other core facilities.

The MVP offers a "one-stop solution" with expert support for EV isolation, purification, biophysical characterization, and high-resolution imaging. Working with the MVP means direct collaboration with scientists experienced in these areas.

Specific research areas and the supporting core facilities include:

- **Molecular Cargo Analysis:**
    - Detection of miRNA, mRNA, and circRNA often utilizes techniques like NanoString nCounter, which can be supported by the Genomics unit.
    - Identification of small metabolites (lipids, small molecules) uses techniques such as NMR, LC-MS, and GC-MS. This is supported by the **Metabolomics Trinity : Metabolic Profiling, Targeted Metabolomics, and Mass Spectrometry Imaging.**
    - **Particle Characterization:**
    -
    - Concentration and distribution measurements (Nanoparticle Tracking Analysis (NTA), Tunable Resistive Pulse Sensing (TRPS), Analytical Ultracentrifugation, Flow Cytometry) are supported by the Flow Cytometry unit.
    - Surface charge (Zeta Potential) and mechanical stiffness (Atomic Force Microscopy (AFM), Optical Tweezers) are also critical characterization aspects.
- **Imaging and Structural Analysis:**
    - High-resolution imaging (Transmission Electron Microscopy (TEM), Cryo-TEM, AFM) and 3D imaging (Focused Ion Beam-Scanning Electron Microscopy (FIB-SEM), Confocal Microscopy) are essentialfor understanding EV structure. The Electron Microscopy and Light Microscopy units provide these capabilities.
    - For spatial mapping of molecules within tissues or cells, Mass Spectrometry Imaging is the only unit providing spatial maps.
- **Functional Studies:**
    - Assessing EV effects on live cells (bioactivity) and tracking cellular interactions and molecular delivery (Fluorescence Microscopy, Flow Cytometry, live-cell imaging) are supported bythe Flow Cytometry and Light Microscopy units.

- **Single Particle Analysis:** Techniques like TRPS, NTA, qNan, and ZetaView enable high-throughput single-particle precision.
