# Supplementary material for: Improving core facility service discovery with an AI assistant grounded in institutional web content
Source: J Biomol Tech. 2026 Jun 27;37(2):40–9. doi: 10.7171/001c.162898 (PMC13313189; doi:10.7171/001c.162898)
Supplement: Supplemental File [file jbt_2026_37_2_162898_347725.pdf]

For every answer, four separate scores were assigned:

1. Correctness and factual grounding (0–10)
    - 0–2: Mostly incorrect or hallucinated; contradicts website information.
    - 3–4: Some correct elements, but key facts wrong or missing; unreliable.
    - 5–6: Largely correct, minor inaccuracies; could be used with human checking.
    - 7–8: Correct and consistent with core facility pages; no obvious errors.
    - 9–10: Fully correct, explicitly grounded in documented services/policies (e.g. correct unit, service type, and constraints).
2. Relevance and routing to cores/contacts (0–10)
    - 0–2: Off-topic, or routes to clearly wrong unit(s); no actionable direction.
    - 3–4: Partially relevant but misses key unit, or mixes irrelevant units.
    - 5–6: Routes to at least one reasonable unit but not all obvious candidates; contact/next-step information incomplete.
    - 7–8: Correct key unit(s), clear indication what each does, and basic next steps (how to start, where to read more).
    - 9–10: Precise routing (all relevant units, none spurious), clear rationale, and explicit next steps and contact paths.
3. Completeness and usefulness (0–10)
    - 0–2: Fragmentary answer; user would still not know what to do.
    - 3–4: Minimal answer; addresses the question but omits important caveats or follow-up steps.
    - 5–6: Provides a usable answer but lacks some details an inexperienced user would need.
    - 7–8: Substantially complete, anticipates obvious follow-up questions (e.g. sample type, typical flow, links).

- 9–10: Fully actionable; includes units, reasoning, key options, and practical guidance tailored to the question type.

4. Style and clarity (0–10)
   - 0–2: Confusing, disorganized, or jargon-heavy; hard to follow.
   - 3–4: Understandable but poorly structured, long, or redundant.
   - 5–6: Clear overall but could be more concise or better structured.
   - 7–8: Well structured (headers/bullets where needed), concise and easy to read for students and PIs.
   - 9–10: Very clear, well-organized, consistent tone; directly usable as user-facing text without editing.
