# Supplementary material for: Improving core facility service discovery with an AI assistant grounded in institutional web content
Source: J Biomol Tech. 2026 Jun 27;37(2):40–9. doi: 10.7171/001c.162898 (PMC13313189; doi:10.7171/001c.162898)
Supplement: Supplemental File [file jbt_2026_37_2_162898_347727.pdf]

Note that actual links and contact names have been deleted in the following chats.

Q1:

Question: My antibody is weak how can it be improved:

Chat: **Summary**

If your antibody has weak binding affinity, it can be improved through molecular engineering and affinity maturation services provided by the Antibody Engineering unit.

Service Details

The unit offers several advanced methods to enhance antibody properties:

- **Affinity Maturation:** The process of improving an antibody's binding strength by introducing mutations and selecting for higher-affinity variants.
- **Structure-Guided Improvement:** Utilizing computational design and structural data to optimize antigen-binding variable regions, often performed during antibody humanization.
- **Single-Cell Sequencing:** Selecting B cells from immunized animals based on specific binding and sequencing their genes to produce high-affinity recombinant monoclonal antibodies.
- **Customized Project Plans:** Each project begins with a personal consultation to define research goals, followed by a plan with clear milestones and data-driven support.

To start a project, please visit the How to Order page.

Scientific Context

Affinity maturation is a process that mimics the natural evolution of B cells. In the lab, researchers create libraries of antibody variants (often by mutating the complementarity-determining regions, or CDRs) and use high-throughput screening (such as FACS or phage

display) to identify clones with a lower dissociation constant (K_D). This results in tighter binding through optimized non-covalent interactions like hydrogen bonds and electrostatic forces.

Actionable Info

For a personal consultation regarding your antibody, please contact the unit head:

- **Head of Unit:**
- **Location:**
- **Staff:**

Q2:

Question: how do I measure the affinity of my antibody:

Chat: **Summary**

To measure the affinity of your antibody, you should contact the Protein Analysis unit. They provide several biophysical techniques to quantify molecular interactions, including Surface Plasmon Resonance (SPR) and Isothermal Titration Calorimetry (ITC).

Service Details

The unit offers the following methods for affinity measurement:

- **Surface Plasmon Resonance (SPR):** Performed on **Biacore S200** or **8K** instruments. This label-free technique measures real-time kinetics, providing on-rate and off-rate constants, as well as the equilibrium dissociation constant.
- **Isothermal Titration Calorimetry (ITC):** Using the **ITC PEAQ**, this is considered the "gold standard" for measuring binding affinity. It directly measures the heat exchange during binding to determine, stoichiometry, enthalpy , and entropy.

- **Microscale Thermophoresis (MST):** Using **Monolith** instruments, this method quantifies interactions in solution by detecting changes in the hydration shell or size of molecules as they move along a microscopic temperature gradient.

Actionable Info

For more information or to schedule a measurement, please contact the unit:

- **Head of Unit:**
- **Location:**
- **Staff:**.

If you are also interested in improving the antibody's affinity, you can consult the Antibody Engineering unit.

<u>Q3:</u>

Question: Measuring RNA expression

Chat: **Summary**

Measuring RNA expression (transcriptomics) is primarily handled by the Genomics units at the Weizmann Institute. Depending on your needs, you can utilize the full-service Genomics: G-INCPM or the hands-on Genomics, Sandbox (LSCF).

Service Details

The units provide a range of technologies for both bulk and single-cell RNA analysis:

- **Bulk RNA-seq (Mars-seq):** A high-throughput, low-input 3’-mRNA-seq method designed for preparing libraries from 1–50 ng of total RNA.
- **Single Cell RNA-seq:** Utilizing **10x Genomics Chromium** (GemCode technology) for high-throughput single-cell 3' transcriptomics.
- **Sequencing Platforms:**

    - **NovaSeqX Plus (Illumina):** Ultra-high-throughput sequencing for large-scale projects.
    - **Aviti (Element Biosciences):** High-quality, flexible benchtop sequencing.
    - **Nextseq 500/550 (Illumina):** Desktop sequencers for transcriptome and whole-genome sequencing.
    - **PacBio Sequel:** Long-read sequencing for capturing full-length transcripts and isoforms.

Bioinformatics Analysis

For the analysis of RNA expression data, you can consult with the following units:

- Bioinformatics: G-INCPM
- Bioinformatics: LSCF

Actionable Info

- **Contact:**
- **Head of Unit:**
- **Staff:**.
- **Project Submission:**

Q4:

Question: what methods can i use to sort cells

Chat: **Summary**

Cell sorting is primarily handled by the Flow Cytometry unit. For single-cell genomic workflows, the Genomics, Sandbox unit provides advanced single-cell multiomics technologies (such as 10x Genomics) which often utilize cells previously sorted by flow cytometry.

Service Details

The Flow Cytometry unit offers several methods and instruments for sorting:

- **Instruments:**
  - **FACSAriaIII:** A four-laser cell sorter.
  - **FACSymphony S6:** A high-parameter cell sorter with 5 lasers.
  - **BioSorter:** A large particle sorter for objects that are too large for standard flow cytometers (e.g., small organisms, large cells).
  - **BD FACSDiscover S8:** Features BD CellView Image Technology for image-based sorting.
- **Sorting Formats:** Cells can be sorted into tubes, plates, or onto slides.
- **Specialized Options:**
  - **Sterile Sorting:** Available for downstream culture or functional assays.
  - **Temperature Control:** Sorting chambers can be cooled or heated as required.
  - **Nozzle Sizes:** Various sizes (70, 85, 100, or 130 microns) are used depending on the required speed and cell size.
- **Sample Preparation:** It is essential to filter all samples (40-50 µm) before sorting to prevent clogging. Cells should be suspended in Calcium and Magnesium-free PBS.

The Genomics, Sandbox unit provides access to **10x Genomics single-cell multiomics** and **spatial transcriptomics**. These technologies allow for advanced single-cell analysis, often following an initial sorting step in the Flow Cytometry unit.

Scientific Context: How it Works

Flow cytometry-based cell sorting, or Fluorescence-Activated Cell Sorting (FACS), works through several key steps:

1. **Hydrodynamic Focusing:** Cells in a liquid suspension are focused into a single-file stream.
2. **Laser Interrogation:** As cells pass through laser beams, they scatter light and emit fluorescence based on markers or dyes.
3. **Droplet Formation:** The stream is vibrated to break into individual droplets, ideally containing one cell each.

4. **Electrostatic Deflection:** Based on the detected signals, a specific electrical charge is applied to the droplet containing the cell of interest. Charged deflection plates then pull these droplets into collection tubes, while uncharged droplets go to waste.

Actionable Information

- **Flow Cytometry:** To schedule a sort or for technical guidance, contact the unit head.
- **Genomics Sandbox:** For single-cell sequencing projects, contact the unit head, . Note that Sandbox equipment requires a workshop for independent use.
- **G-INCPM Projects:** If your research involves large-scale genomic sequencing (e.g., using the NovaSeqX Plus), you may need to Submit a G-INCPM Project.

Q5:

Question: what sorters ar available

Chat: The Flow Cytometry unit provides a variety of specialized sorters categorized by the size and nature of the objects being sorted.

Conventional Cell Sorters

These instruments are designed for particles ranging from **0.5 to 40 µm** and can deposit sorted populations into tubes or plates, often under sterile conditions.

- **FACSAriaIII:** Available in two configurations:
  - **5 lasers:** Can be used with either a violet (405 nm) or a near-UV (375 nm) laser, alongside blue, yellow-green, and red lasers.
  - **4 lasers:** Equipped with violet, blue, yellow-green, and red lasers.
- **FACSymphony S6:** A high-parameter sorter with **5 lasers** (UV 355nm, Violet 405nm, Blue 488nm, Yellow-Green 561nm, and Red 637nm).
- **SORP-FACSAriaII:** Equipped with **5 lasers** (Violet 405nm, Blue 488nm, Yellow-Green 561nm, and Red 640nm).
- **FACSAria FUSION:** A **4-laser** system.

Large Particle Sorter

- **BioSorter:** Specifically designed for larger and/or more fragile objects ranging from **10 to 1,500 µm** (e.g., small organisms or large cells). It features **4 lasers** and can trace fluorescence intensity changes along the entire length of the object.

Imaging-Based Sorter

- **BD FACSDiscover S8:** A cell sorter that incorporates **BD CellView Image Technology**, allowing for sorting based on visual/spatial characteristics of the cells.

Actionable Information

For technical guidance or to schedule a session, please contact the unit staff:

- **Head of Unit:** (Location: Dwek Campus Center, Room 8).
- **Staff:** .

If your sorting is a precursor to single-cell genomics, you may also want to consult the Genomics, Sandbox unit.

Q6.

Question: What sort of metabolites can be found in plants

Chat: Plant metabolites are broadly categorized into primary metabolites, which are essential for growth and development, and secondary (specialized) metabolites, which are involved in defense and environmental interactions. At the Weizmann Institute, these are analyzed across several specialized units.

Summary

Research into plant metabolites is supported by the Metabolic Profiling, Targeted Metabolomics, and Mass Spectrometry Imaging units. These facilities provide comprehensive analysis of small molecules, lipids, and specialized plant compounds.

Service Details

- **Metabolic Profiling:** This unit provides non-targeted profiling of three main groups:
  - **Core Metabolites:** Essential compounds such as sugars, organic acids, and amino acids.
  - **Lipids:** Comprehensive lipidome profiling including free fatty acids, phospholipids, sphingolipids, and triacylglycerols.
  - **Specialized Metabolites:** Semi-polar compounds such as pigments (e.g., betalains), flavonols, and steroidal metabolites (e.g., alkaloids in *Solanum*).
  - **Instrumentation:** Utilizes UPLC/HPLC coupled with Q-Exactive, QTOF, LTQ XL Orbitrap, and LC-Triple Quad Mass Spectrometers.
- **Targeted Metabolomics:** Focuses on the precise quantification of specific known metabolites.
- **Mass Spectrometry Imaging:** This is the **only unit** providing spatial maps of metabolites, allowing researchers to see exactly where specific molecules are localized within plant tissues.
- **Bioinformatics Support:** For the analysis of complex metabolomics data, services are available through both Bioinformatics: LSCF and Bioinformatics: G-INCPM.

Scientific Context

Plants produce a vast array of organic compounds:

- **Primary Metabolites:** Universal molecules like carbohydrates (glucose, starch), amino acids, and nucleotides that support core physiological processes.
- **Secondary Metabolites:** Often species-specific compounds such as phenolics (flavonoids, tannins), terpenoids (essential oils, carotenoids), and alkaloids (nicotine, caffeine). These are crucial for UV protection, signaling, and defense against herbivores.

Actionable Information

For project consultations or to start a new analysis:

- **General Inquiries:** Contact the units via their respective "About" pages linked above.
- **G-INCPM Projects:** Submit a G-INCPM Project for bioinformatics or specialized profiling services.
- **Pricing:** Please contact the relevant unit directly for current service rates and project quotes.

Q7

Question: which core facilities support extracellular vesicle research and why

Chat: Research into extracellular vesicles (EVs) at the Weizmann Institute is primarily supported by the **Multidisciplinary Vesicle Program (MVP)**, which serves as a central hub for vesicle expertise, isolation, and characterization.

Summary of Supporting Units

- **Primary Hub:** The **Multidisciplinary Vesicle Program (MVP)** provides integrated scientific and analytical expertise, including isolation workflows (SEC purification), biophysical characterization, and nanoparticle analytics.
- **Analysis & Sorting: Flow Cytometry** is used for measuring particle concentration, size distribution, and assessing EV bioactivity or cellular uptake.
- **Omics & Composition:**
  - **Lipids & Metabolites:** For analyzing the small molecule and lipid cargo of EVs, services are provided by **Metabolic Profiling**, **Targeted Metabolomics**, and **Mass spectrometry imaging**. Note that **Mass spectrometry imaging** is the only unit providing spatial maps of these molecules.
  - **Proteomics: Protein Profiling: G-INCPM** handles high-sensitivity protein identification and quantification.
  - **Genomics: Genomics: G-INCPM** provides RNA-Seq and other transcriptomic services to detect miRNA, mRNA, and circRNA within vesicles.

- **Imaging:** High-resolution visualization (TEM, Cryo-TEM, AFM) and mechanical stiffness measurements are supported by the **MICC Cell Observatory**.
- **Data Analysis:** General bioinformatic support for EV-related datasets is available through both **Bioinformatics: G-INCPM** and **Bioinformatics: LSCF**.

Why Extracellular Vesicles are Researched

Extracellular vesicles, including exosomes and microvesicles, are naturally secreted nanoscale particles that facilitate intercellular communication. They are of high research interest because:

1. **Molecular Carriers:** They transfer proteins, lipids, and nucleic acids between cells, influencing the behavior of recipient cells.
2. **Biomarkers:** They reflect the physiological or pathological state of their parent cells, making them ideal candidates for non-invasive diagnostics (liquid biopsies) in diseases like cancer and neurodegeneration.
3. **Therapeutic Delivery:** Due to their biocompatibility and natural targeting potential, they are being developed as biologically inspired delivery systems for drugs and gene therapy.

Actionable Information

- **Consultation:** MVP offers introductory meetings and preliminary discussions free of charge to help design isolation and characterization workflows.
- **G-INCPM Projects:** For services involving G-INCPM units (Genomics, Protein Profiling, or Bioinformatics), you can Submit a G-INCPM Project.
- **Contact:**
