# Supplementary material for: Improving core facility service discovery with an AI assistant grounded in institutional web content
Source: J Biomol Tech. 2026 Jun 27;37(2):40–9. doi: 10.7171/001c.162898 (PMC13313189; doi:10.7171/001c.162898)
Supplement: Supplemental File [file jbt_2026_37_2_162898_347728.pdf]

```python
# list_store_docs.py
import argparse
from google import genai

def parse_args():
    parser = argparse.ArgumentParser(
        description="List documents in a Gemini File Search store."
    )
    parser.add_argument(
        "-store",
        "--store",
        required=True,
        help="Full File Search store name (e.g. fileSearchStores/xxxx).",
    )
    parser.add_argument(
        "-api",
        "--api",
        required=True,
        help="Path to a text file containing the Gemini API key.",
    )
    return parser.parse_args()

def read_api_key(path: str) -> str:
    with open(path, "r", encoding="utf-8") as f:
        return f.read().strip()

def main():
    args = parse_args()
    api_key = read_api_key(args.api)
    client = genai.Client(api_key=api_key)

    print(f"Documents in store {args.store}:")
    for doc in client.file_search_stores.documents.list(
        parent=args.store
    ):
        print(f"- {doc.name} | display_name={doc.display_name}")

if __name__ == "__main__":
    main()
```
